# Great ape Y Chromosome and mitochondrial DNA phylogenies reflect subspecies structure and patterns of mating and dispersal

**DOI:** 10.1101/gr.198754.115

**Published:** 2016-04

**Authors:** Pille Hallast, Pierpaolo Maisano Delser, Chiara Batini, Daniel Zadik, Mariano Rocchi, Werner Schempp, Chris Tyler-Smith, Mark A. Jobling

**Affiliations:** 1 Department of Genetics, University of Leicester, Leicester LE1 7RH, United Kingdom;; 2 Institute of Molecular and Cell Biology, University of Tartu, Tartu 51010, Estonia;; 3 Department of Biology, University of Bari, 70124 Bari, Italy;; 4 Institute of Human Genetics, University of Freiburg, 79106 Freiburg, Germany;; 5 Wellcome Trust Sanger Institute, Wellcome Trust Genome Campus, Hinxton, Cambridge CB10 1SA, United Kingdom

## Abstract

The distribution of genetic diversity in great ape species is likely to have been affected by patterns of dispersal and mating. This has previously been investigated by sequencing autosomal and mitochondrial DNA (mtDNA), but large-scale sequence analysis of the male-specific region of the Y Chromosome (MSY) has not yet been undertaken. Here, we use the human MSY reference sequence as a basis for sequence capture and read mapping in 19 great ape males, combining the data with sequences extracted from the published whole genomes of 24 additional males to yield a total sample of 19 chimpanzees, four bonobos, 14 gorillas, and six orangutans, in which interpretable MSY sequence ranges from 2.61 to 3.80 Mb. This analysis reveals thousands of novel MSY variants and defines unbiased phylogenies. We compare these with mtDNA-based trees in the same individuals, estimating time-to-most-recent common ancestor (TMRCA) for key nodes in both cases. The two loci show high topological concordance and are consistent with accepted (sub)species definitions, but time depths differ enormously between loci and (sub)species, likely reflecting different dispersal and mating patterns. Gorillas and chimpanzees/bonobos present generally low and high MSY diversity, respectively, reflecting polygyny versus multimale–multifemale mating. However, particularly marked differences exist among chimpanzee subspecies: The western chimpanzee MSY phylogeny has a TMRCA of only 13.2 (10.8–15.8) thousand years, but that for central chimpanzees exceeds 1 million years. Cross-species comparison within a single MSY phylogeny emphasizes the low human diversity, and reveals species-specific branch length variation that may reflect differences in long-term generation times.

Patterns of dispersal and mating are key factors in determining the distribution of genetic diversity within species (Dieckmann et al. 1999; Storz 1999). Among primates (Dixson 2013), male-biased dispersal and female philopatry are generally the norm; and in this context, our closest living relatives, the African apes, present an anomalous pattern in which females migrate out of their natal communities and join neighboring groups. This is most marked in chimpanzees and bonobos, which show multimale–multifemale mating structures in which females mate with most of the unrelated males in their communities. In gorillas, which show primarily polygynous mating structures in which a single dominant male fathers most of the offspring, females commonly disperse when they mature, whereas males either leave or remain until they have an opportunity to attain dominant status in the group (Harcourt and Stewart 2007). These observations have suggested that male philopatry may be an ancestral feature of African apes and humans (Wrangham 1987). The remaining great apes, the Asian orangutans, present a distinct social organization in which the sexes are spatially separate and occupy large individual ranges, and the limited observational data have suggested male-biased dispersal (Delgado and van Schaik 2000).

Like behavioral ecology, DNA analysis can provide additional evidence about dispersal and mating patterns and their effects, and here, comparisons of biparentally inherited sequences with uniparentally inherited segments of the genome are potentially useful. Autosomal analysis has typically focused on analysis of short tandem repeats (STRs) (e.g., Becquet et al. 2007; Nater et al. 2013; Fünfstück et al. 2014), with increasing numbers of whole-genome sequences recently becoming available (Prado-Martinez et al. 2013; Xue et al. 2015) and providing a rich picture of population structure and demographic history. Maternally inherited mitochondrial DNA (mtDNA) has also been widely exploited, progressing from sequencing of the hypervariable regions (Fischer et al. 2006) to the maximum possible resolution of the whole molecule (Hvilsom et al. 2014). Diversity of the male-specific region of the Y Chromosome (MSY), however, has been much less exploited in studies of great apes. Several studies have applied MSY-specific STRs, discovered by assaying the orthologs of human Y-STRs for amplifiability and polymorphism (Erler et al. 2004). The resulting haplotypes are variable in all great ape populations and have been useful in revealing aspects of sex-biased dispersal in bonobos (Eriksson et al. 2006), chimpanzees (Schubert et al. 2011; Langergraber et al. 2014), western lowland gorillas (Douadi et al. 2007; Inoue et al. 2013), and orangutans (Nater et al. 2011; Nietlisbach et al. 2012).

However, despite their highly variable nature and lack of ascertainment bias, Y-STRs suffer from problems of allele homoplasy and cannot be reliably used to understand distant relationships between MSY types (Wei et al. 2013; Hallast et al. 2015). In humans, their utility has been enhanced by combining them with a robust MSY phylogeny of haplogroups based on slowly mutating single-nucleotide polymorphisms (SNPs) (Jobling and Tyler-Smith 2003). A few great ape MSY SNPs have been identified by small-scale resequencing studies. Analysis of ∼3 kb of MSY DNA in 101 chimpanzees, seven bonobos, and one western lowland gorilla (Stone et al. 2002) yielded 23 SNPs within the *Pan* genus, defining subspecies-specific lineages among chimpanzees and suggesting higher diversity than among humans. Another study identified six SNPs and one indel among orangutan MSY sequences (Nietlisbach et al. 2010).

In principle, next-generation sequencing (NGS) offers the possibility of greatly increasing the number of useful MSY SNPs among great apes and providing highly resolved phylogenies in which branch lengths reflect evolutionary time. This is illustrated by the case of mountain gorillas, for which a MSY phylogeny based on NGS data shows extremely low diversity (Xue et al. 2015). Such phylogenies would be useful tools for studying great ape population structure, sex-biased behaviors, the dynamics of MSY mutation processes, and lineage-specific effects of male-biased mutation. However, two obstacles exist: the lack of a MSY reference sequence for most great ape species, and the high degree of inter-specific structural divergence of the Y Chromosome.

The human MSY reference sequence is of particularly high quality and was derived by the painstaking assembly of a bacterial artificial chromosome tiling path largely from the DNA of one man, followed by Sanger sequencing (Skaletsky et al. 2003). A similar approach has been applied in the chimpanzee (Hughes et al. 2010) and rhesus macaque (Hughes et al. 2012), so these reference sequences provide reliable starting points for analyzing intraspecific variation among primates. Unfortunately, no such approach has yet been applied to bonobos, gorillas or orangutans, and indeed for these species the reference genomes were derived from female individuals (Locke et al. 2011; Prüfer et al. 2012; Scally et al. 2012) to maximize X-Chromosomal coverage.

Fluorescence in situ hybridization (FISH) analysis (Archidiacono et al. 1998; Gläser et al. 1998) of the Y Chromosome in great apes and other primates has given a broad-scale view of its cytogenetic evolution and revealed a remarkably high degree of interspecies divergence in sequence content and organization, in contrast to the general cytogenetic stability of the rest of the genome (Yunis and Prakash 1982). This has been confirmed at the sequence level by a comparison (Hughes et al. 2012) of the human, chimpanzee, and rhesus macaque reference MSY assemblies, in which the euchromatic regions vary in their sizes (25.8, 22.9, and 11.0 Mb, respectively) and representations of different sequence classes.

Given these difficulties, we chose to apply an anthropocentric approach to defining and sequencing orthologous regions of the MSY in great apes. Previously (Batini et al. 2015; Hallast et al. 2015), we used targeted sequence-capture to obtain and sequence 4.43 Mb of MSY in each of 448 human males at a mean coverage of 44×. In the same experiments, we included 19 great ape males, capturing MSY sequences efficiently based on a human reference sequence design and providing useful ancestral state information for our 13,261 human MSY SNPs. Here, we focus on these great ape sequences, which include species-specific deletions and duplications, but retain between 2.61 and 3.80 Mb (depending on species) of human-orthologous MSY material for analysis. We combine these data with MSY sequences extracted from the published whole genomes of 24 other great ape males (Prado-Martinez et al. 2013; Xue et al. 2015) to yield a total sample of 19 chimpanzees, four bonobos, 14 gorillas, and two Bornean and four Sumatran orangutans. We construct phylogenies using the discovered MSY variants and compare these with phylogenies based on whole mtDNA sequences in the same individuals, estimating the time-to-most-recent common ancestor (TMRCA) for key nodes in both cases. We use the observed differences between loci and (sub)species to provide insights into the effects of different dispersal and mating patterns.

## Results

**Figure 1.**
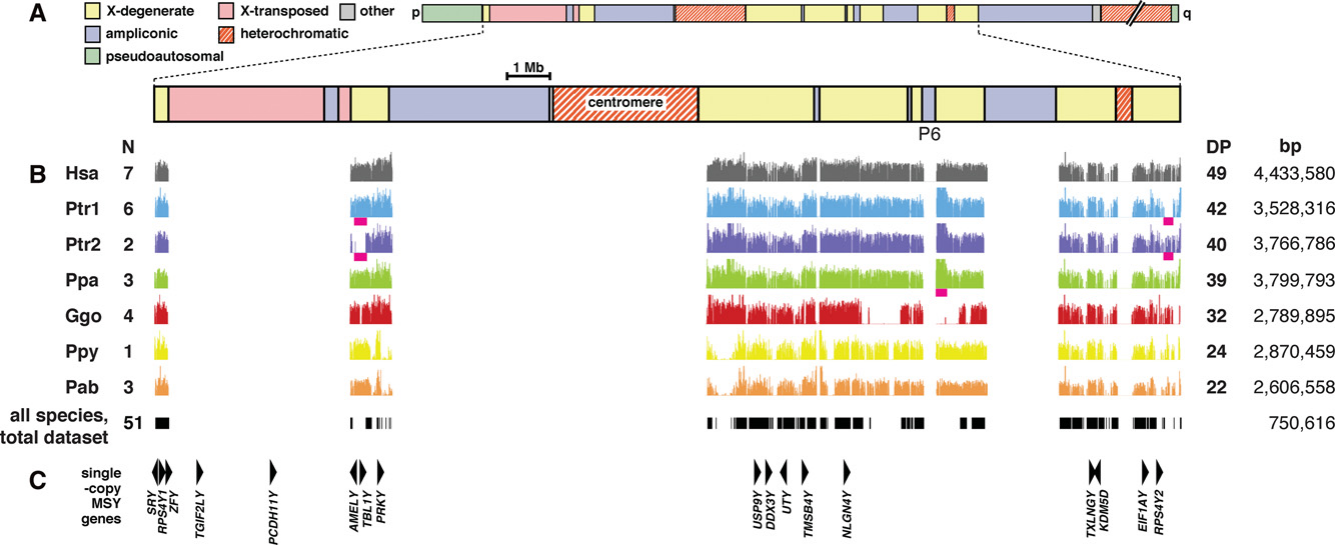
Location and extent of sequenced great ape MSY-orthologous regions compared to the human reference sequence. (*A*) Schematic representation of the human Y Chromosome (Skaletsky et al. 2003) showing blocks of different sequence classes. (*B*) The analyzed subregions of MSY, shown as plots of read depth against chromosome position. Note that the order and orientation of MSY sequences in the great apes is not necessarily the same as that in the human reference sequence. In each plot, the *y*-axis ranges from zero to 150×. Sample size (N) for each species is given to the *left*, and mean depth (DP) and the extent of sequence obtained (bp) to the *right*. (Hsa) human; (Ptr) chimpanzee; (Ppa) bonobo; (Ggo) gorilla; (Ppy) Bornean orangutan; (Pab) Sumatran orangutan. Chimpanzees carry two distinct structural variant sequences (Ptr1 and 2) differing by insertion/deletions highlighted by magenta bars. Similarly highlighted is a *Pan*-specific duplication that extends palindrome P6. *Below* the species plots, black bars indicate sequenced regions shared across all 51 males (43 great apes and seven humans as a representative subset from the 448 sequenced samples, plus one haplogroup A00 human) (Hallast et al. 2015; Karmin et al. 2015), totaling 750,616 bp, and used in constructing the cross-species phylogeny shown in Figure 4 (see below). (*C*) Locations of single-copy MSY genes (Skaletsky et al. 2003; Bellott et al. 2014) shown as triangles (not drawn to scale) pointing in the direction of transcription.

To obtain MSY sequence data from great apes, we included 19 males (eight chimpanzees, three bonobos, four gorillas, and one Bornean and three Sumatran orangutans) (Supplemental Table S1) together with 448 human males in a sequence-capture experiment based on a human-reference-sequence design, as described previously (Hallast et al. 2015). In each of the human samples, this approach yielded 4.43 Mb of analyzable MSY sequence, excluding the ampliconic and X-transposed regions (Skaletsky et al. 2003) of the chromosome (Fig. 1).

We were also interested to compare MSY diversity with that of other components of the great ape genomes. We had included within the human-based sequence capture design 11,500 120-nt capture baits distributed quasi-randomly across the genome (see Methods) in order to provide a general picture of genome-wide diversity. In the great ape samples, we analyzed the orthologs of these sequences to assess autosomal and X-Chromosomal intra-specific variation. Finally, we also sequenced the whole mitochondrial genomes of all 19 great ape individuals.

To increase the number of great ape samples analyzed, we also extracted the genomic regions described above (where possible) from the published whole-genome sequences of an additional 92 independent individuals (Supplemental Table S1; Prado-Martinez et al. 2013; Xue et al. 2015), including 24 males. This led to a total male sample size of 43.

### Confirming subspecies status by autosomal PCA

**Figure 2.**
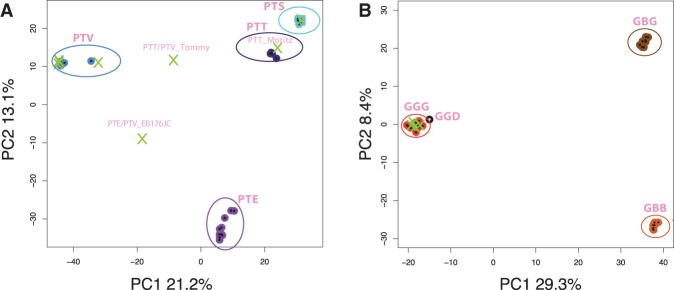
Confirmation of subspecies status in chimpanzees and gorillas using PCA of autosomal SNPs. PCA plots based on autosomal SNP variation: (*A*) the eight chimpanzees sequenced here (crosses), plus 25 published individuals (Prado-Martinez et al. 2013) of known subspecies status (circles); (*B*) the four gorillas sequenced here (crosses), plus 44 published individuals (Prado-Martinez et al. 2013; Xue et al. 2015) of known subspecies status (circles). (PTT) *Pan troglodytes troglodytes*; (PTS) *P. t. schweinfurthii*; (PTE) *P. t. ellioti*; (PTV) *P. t. verus*; (GGG) *Gorilla gorilla gorilla*; (GGD) *G. g. diehli*; (GBB) *G. beringei beringei*; (GBG) *G. b. graueri*.

In order to clarify and confirm the subspecies status of the 19 male samples sequenced here, we carried out principal component analysis (PCA) of autosomal SNP variation (∼10,000–48,000 variable sites, depending on species) (Supplemental Table S2) together with the previously published samples (both male and female) in which subspecies designation was known. Based on this analysis (Fig. 2; Supplemental Figure S1), 17 of our 19 sequenced individuals lie within known subspecies clusters, thus confirming their subspecies status. For chimpanzees, three of the four subspecies (*Pan troglodytes verus*, *P. t. troglodytes*, and *P. t. schweinfurthii*) are represented in our sample (Fig. 2A), and for gorillas, all four of our individuals (Fig. 2B) belong to the western lowland subspecies (*Gorilla gorilla gorilla*). Two of the sequenced chimpanzees lie midway between clusters in the PCA (Fig. 2A), suggesting recent inter-subspecies hybridization in their ancestry (Tommy: *P. t. verus*/*P. t. troglodytes* hybrid; EB176JC: *P. t. verus*/*P. t. ellioti* hybrid) (Supplemental Fig. S2). This conclusion is supported by model-based estimation of ancestry (Supplemental Figs. S3, S4).

### Characteristics of great ape MSY sequences

Following mapping of sequence reads to the human reference and filtering (see Methods), we obtained orthologous MSY sequence data for all 19 great ape males. Despite the anthropocentric design, sequence capture worked well for all species, although orangutans show lower read depths than other species (Fig. 1B), possibly reflecting reduced capture efficiency due to the relatively high sequence divergence from the human reference. As expected, the final extent of orthologous MSY sequence is reduced in the great ape species compared to humans, most likely as a result of deletions in the great ape lineages (Fig. 1B). Bonobos show the greatest MSY-orthologous sequence content (3.80 Mb), whereas most chimpanzees (“Ptr1” in Fig. 1B) show a somewhat lower level (3.53 Mb), and gorillas (2.77 Mb) and orangutans (2.87 Mb [Bornean]; 2.61 Mb [Sumatran]) lower still. A low-resolution survey of sequence depth suggests that chimpanzees and bonobos carry a duplication of a 207-kb long-arm-orthologous segment, representing a *Pan*-specific extension of the P6 palindrome compared with all other species, and consistent with the chimpanzee MSY reference sequence (Hughes et al. 2010). Comparison among individuals within species reveals a generally low level of large-scale insertion/deletion polymorphism. The only striking example is seen among the chimpanzees. Two individuals (Tommy and Moritz, possessing the structure designated “Ptr2” in Fig. 1B) carry a large deletion compared to the other six (Ptr1): This is equivalent to 270 kb of human short-arm-orthologous sequence, but mapping to the chimpanzee MSY reference sequence suggests that the deletion's actual size is ∼439 kb (Supplemental Fig. S5). At the same time, these two individuals retain a 167-kb segment of long-arm-orthologous material that is absent from the majority of chimpanzees (Fig. 1B).

There has been much debate about the functional importance of human single-copy MSY genes, so the retention or otherwise of these genes among great apes is a matter of interest. At the gross scale, patterns of presence or absence of the 15 XY-homologous single-copy genes illustrated in Figure 1C (excluding the human-specific X-transposed-region genes *TGIF2LY* and *PCDH11Y*) are generally as expected from previous studies (Cortez et al. 2014) in all 19 individuals studied. There are two exceptions: First, we find the *AMELY* gene to be present in all orangutans, in contrast to the published report of its absence (Cortez et al. 2014); and second, although *AMELY* and *TBL1Y* are present in most chimpanzees, they are absent from the Ptr2 structure since they lie within the 270-kb deletion.

### MSY diversity in great apes and comparison with other parts of the genome

We merged MSY sequences in the 19 males analyzed here with equivalent sequences from the 24 published samples, and identified SNPs. Despite the small sample sizes, this yielded a large number of variants: 1262 MSY SNPs among gorillas; 2476 among orangutans; 3284 among bonobos; and 12,208 among chimpanzees. Table 1 summarizes sequence diversity estimates for different parts of the genome in the various species and subspecies (see also Supplemental Table S2). This confirms generally low MSY diversity, with the exception of bonobos and central chimpanzees. Levels of autosomal heterozygosity are not clearly correlated with MSY diversity, and ratios of mtDNA:MSY nucleotide diversity differ widely. For example, in humans, this ratio is ∼20, but it varies from less than three in central chimpanzees, to more than 400 in Sumatran orangutans. Together, this suggests that the uniparentally inherited loci have been strongly affected by drift (or selection) and probably by differing mating and dispersal patterns.

**Table 1.**
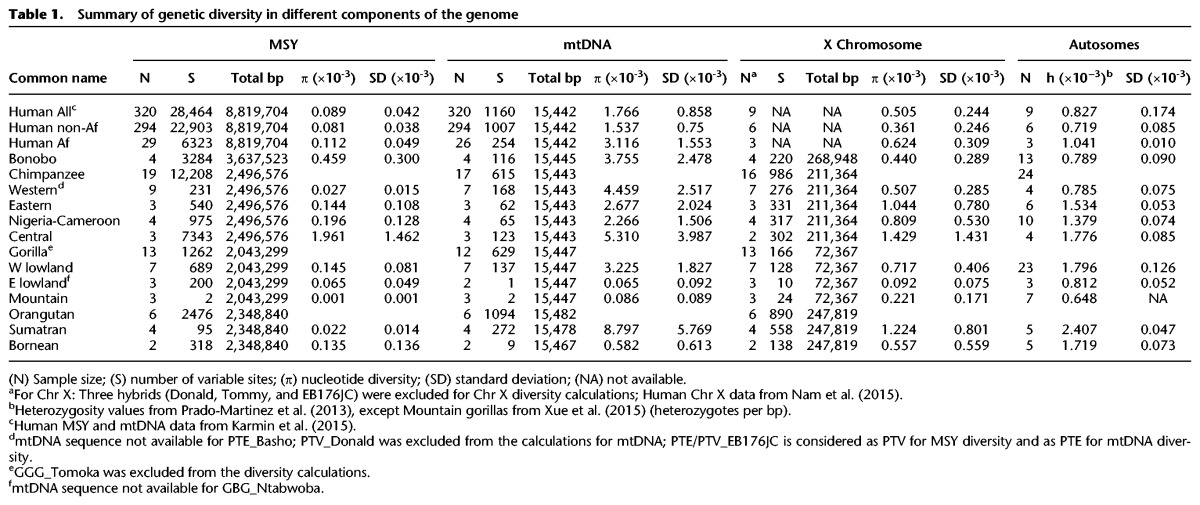
Summary of genetic diversity in different components of the genome

### Characteristics of the MSY phylogenies in great ape species and comparison with mtDNA

In order to better understand the different histories of MSY and mtDNA sequences in each (sub)species, we constructed maximum-parsimony trees based on the SNPs identified within each locus. For the mtDNA phylogeny, we considered only those (male) individuals for which we also had MSY sequence data.

#### Orangutans

**Figure 3.**
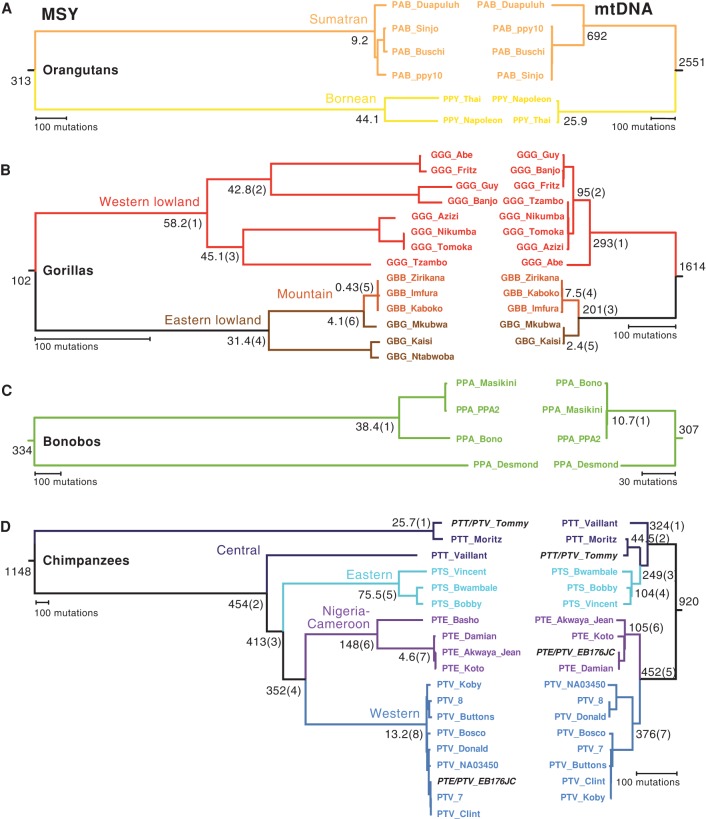
MSY and mtDNA phylogenies in great ape species. MSY (*left*) and mtDNA (*right*) phylogenies: (*A*) orangutans; (*B*) gorillas; (*C*) bonobos; (*D*) chimpanzees. Note that not all phylogenies are to the same mutational scale, which is indicated in each case by a scale bar. Point estimates of TMRCA are given adjacent to selected nodes (95% HPD intervals are available in Table 2); numbers in parentheses highlight specific nodes discussed elsewhere. Species/subspecies are indicated, and names of individuals are given at the tips of branches, as listed in Supplemental Table S1. (PAB) *Pongo abelii*; (PPY) *P. pygmaeus*; (GGG) *Gorilla gorilla gorilla*; (GBB) *G. beringei beringei*; (GBG) *G. b. graueri*; (PPA) *Pan paniscus*; (PTT) *Pan troglodytes troglodytes*; (PTS) *P. t. schweinfurthii*; (PTE) *P. t. ellioti*; (PTV) *P. t. verus*. The two chimpanzee cross-subspecies hybrids are indicated by black italic type; despite his hybrid status, Tommy has both MSY and mtDNA sequences characteristic of central chimpanzees (PTT), whereas EB176JC carries a typically western (PTV) MSY and a Nigeria-Cameroon (PTE) mtDNA sequence. Separate PCA analysis of X-Chromosomal SNPs shows that the X Chromosome of EB176JC clusters with *P. t. ellioti* X Chromosomes (Supplemental Fig. S2).

The four Sumatran orangutan MSY sequences (Fig. 3A) form a shallow phylogeny with a very recent TMRCA of 9.2 (7.2–11.6) thousand years ago (KYA) (Table 2); in contrast, TMRCA for the two Bornean orangutan sequences is 44.1 (37.4–51.5) KYA. Although the sample sizes are too small to draw firm conclusions, this difference reflects neither the picture of autosomal heterozygosity (Prado-Martinez et al. 2013), which is significantly higher in Sumatran orangutans, nor the mtDNA phylogeny (Fig. 3A), in which the Sumatran species shows a deep-rooting node (TMRCA 692 [592–798] KYA), with very little depth in the Bornean species (25.9 [8.9–47.2] KYA). The age of the node separating the MSY sequences of the two orangutan species is 313 (277–353) KYA, and that for mtDNA is 2551 (2354–2754) KYA. Species divergence time estimates based on whole-genome sequences (Locke et al. 2011) are 400 KYA from SNP frequency spectra, and 334 ± 145 KYA from a coalescent-based approach; our MSY-based estimate is consistent with these estimates.

**Table 2.**
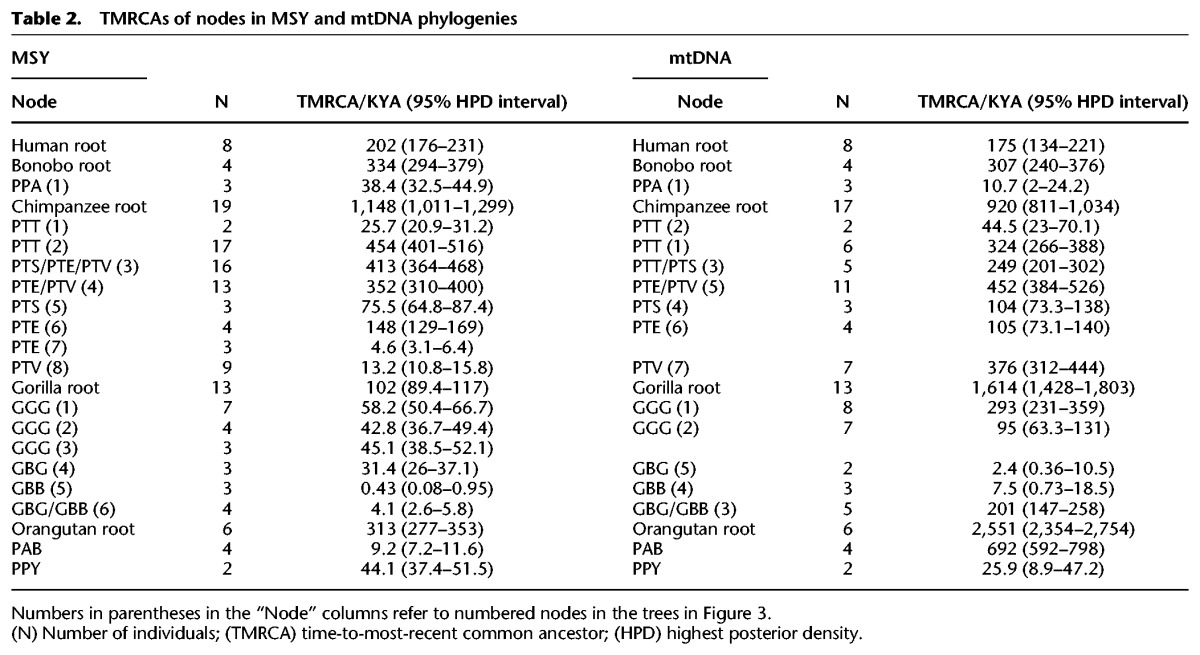
TMRCAs of nodes in MSY and mtDNA phylogenies

#### Gorillas

The gorilla MSY phylogeny (Fig. 3B) also shows clear separation of the two species. The seven western lowland gorilla (*G. g. gorilla*) MSY sequences have a TMRCA of 58.2 (50.4–66.7) KYA, considerably younger than the human Y phylogeny which, when the ancient haplogroup A00 is included, has a TMRCA of 202 (176–231) KYA (this estimate is consistent with a published estimate based on a larger sample size, once differences in mutation rate are accounted for) (Table 2; Karmin et al. 2015). However, among the gorilla sequences, two internal nodes date to >40 KYA. Among the eastern *G. beringei* individuals, the three mountain gorillas (*G. b. beringei*) present very low MSY diversity, as previously noted (Xue et al. 2015), whereas eastern lowland gorillas (*G. b. graueri*) show a TMRCA of 31.4 (26–37.1) KYA. One of the eastern lowland gorillas, GBG_Mkubwa, has an MSY sequence that is quite closely related to those of the mountain gorillas. For gorillas as a whole, the age of the node that separates *G. gorilla* from *G. beringei* is 102 (89.4–117) KYA. Estimates of western–eastern (i.e., interspecies) divergence times from whole-genome sequence data vary widely (Scally et al. 2012; McManus et al. 2015; Xue et al. 2015), but it is generally agreed that exchange of migrants between the emerging western and eastern species continued until quite recently (Mailund et al. 2012), and possibly up until 20 KYA (Xue et al. 2015). The broad topological features of the mtDNA tree (Fig. 3B) and the distribution of (sub)species, are, with the exception of GBG_Mkubwa, similar to those of the MSY tree. The major difference is in time depth: The species split is 1.61 million years ago (MYA), and the *G. gorilla* and *G. beringei* TMRCAs are, respectively, 293 KYA and 201 KYA. This difference between the time depths of maternal and paternal lineages is likely a reflection of male-biased dispersal among gorillas.

#### Bonobos

The four bonobo MSY sequences are phylogenetically distinct from those of chimpanzees (Supplemental Fig. S6). Despite the low autosomal nucleotide diversity in this species (Table 1), the MSY phylogeny (Fig. 3C) contains a remarkably deep node with TMRCA 334 (294–379) KYA, as well as a younger node with TMRCA 38.4 (32.5–44.9) KYA. Three of the four mtDNA sequences (Fig. 3C) are highly similar, differing only by two variants, whereas the third (in Desmond, the same individual who carries the ancient MSY lineage) is highly diverged, contributing to an mtDNA TMRCA of 307 (240–376) KYA.

#### Chimpanzees

MSY sequences among the chimpanzees show imperfect phylogenetic coherence with subspecies status (Fig. 3D). Western chimpanzees (*P. t. verus*) present a very shallow phylogeny with a very young TMRCA of 13.2 (10.8–15.8) KYA. Three of the four Nigeria–Cameroon (*P. t. ellioti*) sequences also form a shallow phylogeny, with a fourth contributing a deep-rooting branch resulting in a TMRCA of 148 (129–169) KYA. The three Eastern chimpanzee sequences (*P. t. schweinfurthii*) have an intermediate TMRCA of 75.5 (64.8–87.4) KYA. The most remarkable feature of the phylogeny relates to the central chimpanzee (*P. t. troglodytes*) sequences, which form a paraphyletic group within the tree. One MSY sequence (in Vaillant) lies basal to the other species, contributing to a TMRCA for this part of the tree of 454 (401–516) KYA. However, the remaining two sequences (in Tommy and Moritz) belong to a very deep branch, contributing to a remarkably ancient overall chimpanzee TMRCA of 1148 (1011–1299) KYA. These two sequences also carry the “Ptr2” structural MSY variant shown in Figure 1B, but are not themselves very closely related, showing a pairwise TMRCA of 25.7 (20.9–31.2) KYA. In the mtDNA phylogeny (Fig. 3D), chimpanzee subspecies are also phylogenetically coherent, as has been noted before (Bjork et al. 2011; Prado-Martinez et al. 2013), but as in the MSY phylogeny, the central subspecies forms a paraphyletic group. In our mtDNA phylogeny, the overall TMRCA is 920 (811–1034) KYA.

#### Cross-species comparison

**Figure 4.**
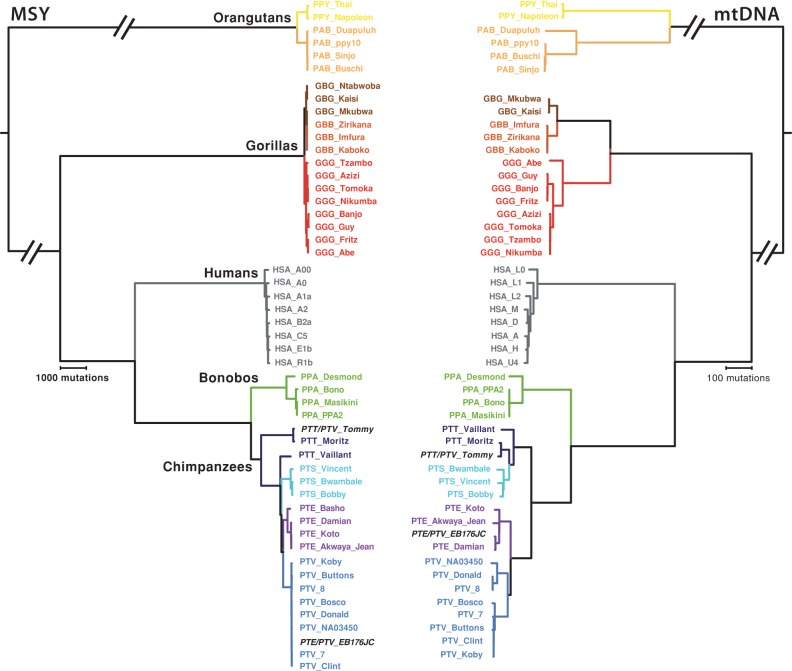
Cross-species MSY and mtDNA phylogenies. Species/subspecies names and names of great ape individuals are given at the tips of branches as in Figure 3. For each human sample, “HSA” (*Homo sapiens*) is followed by the MSY or mtDNA haplogroup name, as listed in Supplemental Table S1. For both MSY and mtDNA, the orangutan branches are truncated for display purposes.

Finally, to give an overview of the relative depths and topologies of MSY phylogenies, we present a cross-species tree based on the 750,616 bp of shared orthologous sequence in Figure 4. The topology of the *Pongo*, *Gorilla*, *Pan*, and *Homo* MSY clades is as expected from other genetic and nongenetic data, but among species, the depths and topologies are markedly different. Set in this context, the human MSY phylogeny appears very shallow, even though it includes the most ancient known lineage (haplogroup A00), with the relationships between haplogroups barely discernible at this scale. The cross-species tree also emphasizes the very low MSY diversity in orangutans and gorillas and the contrasting high diversity in bonobos and chimpanzees. Considering the MSY and mtDNA phylogenies together, of all the great ape species, the combination that most closely resembles that of humans is in the western lowland gorillas. Taken at face value, this might argue against a long human history of multimale–multifemale mating.

Using the orangutans as an outgroup, it is also possible to make a meaningful comparison of tip-to-root mutational lengths of the MSY tree in the other species. These vary considerably: Gorillas have the greatest length (mean of 9393 [SD 24] mutations), followed by bonobos (9078 [SD 47]), chimpanzees (8910 [SD 33]), with humans showing the shortest length (8042 [SD 16]). The African great apes thus show, respectively, 16.8%, 12.9%, and 10.8% longer mean branch lengths than humans. This appears to reflect the increasing generation times from gorillas (20 yr) via chimpanzees (24 yr) to humans (30 yr) (Fenner 2005; Langergraber et al. 2012). However, using the same approach for the mtDNA tree, a different pattern is obtained with the greatest length in chimpanzees (1413 [SD 16]), followed by bonobos (1346 [SD 1]), humans (1246 [SD 7]), and gorillas (1105 [SD 5]). This disparity between MSY and mtDNA could reflect different generation times between the sexes and/or different mutation processes between the nuclear and mitochondrial systems.

## Discussion

### Drawbacks of anthropocentric sequencing approach

In this study, we have taken an anthropocentric approach to great ape sequence capture and read mapping, using the human reference sequence as the basis for both. This has the advantage of simplicity and avoids the problems of missing reference sequences for some species but has some disadvantages. Sequences present in great apes, but absent in humans, will be neither captured nor mapped. However, this is unlikely to introduce bias into the structures of MSY phylogenies, and in any case, the human-chimpanzee reference sequence comparison, at least, indicates only minor differences in the content of the X-degenerate class of MSY sequences (Hughes et al. 2010). Also, the low proportion of recurrent mutations we observe in the species phylogenies (Supplemental Table S2) suggests that hidden structural variants such as duplications are not leading to a high number of errors.

A more serious potential problem is differential capture of nonhuman sequences, depending on their degree of divergence from the human reference—greater average divergence may generally reduce capture efficiency, and locally highly diverged sequences may not hybridize efficiently with baits during capture, and therefore might be lost from the final sequence data set. We see a possible effect of this in the mean read-depth (Fig. 1B), which decreases with expected average divergence from the human sequence. We can estimate the observed average divergence in our MSY sequences between humans and the great ape species; these are 1.43% (chimpanzee), 1.48% (bonobo), 1.95% (gorillas), and 4.34% (orangutans) (Supplemental Table S3). As expected for MSY, these figures are higher than those reported for autosomal DNA, i.e., 1.37% (Scally et al. 2012), 1.3% (Prüfer et al. 2012), 1.75%, and 3.40% (Scally et al. 2012), respectively, but not greatly so. Based on the chimpanzee-human reference sequence comparison, which finds a divergence of 1.7% (Hughes et al. 2010), our value is an underestimate. Taken together, this suggests that lower sequence capture efficiency may have led to artificially shortened interspecies branch lengths and lower TMRCAs. However, we do not expect it to have affected tree topologies or intraspecific variation.

To investigate the possible bias introduced by sequence capture, we compared data obtained from this approach with data from whole-genome sequencing, which should lack any such bias (Supplemental Text; Supplemental Tables S3, S4). Despite differences due to, for example, read depth and read length, the two data sets behave similarly, as judged by divergence from humans and the mean number of variants per species.

We also note that mapping to the human reference has led us to apply strict and therefore conservative filters for missing data, which may lead to underestimation of variants. Consistent with this, when chimpanzee sequences are mapped to the chimpanzee reference sequence (Supplemental Fig. S7; Supplemental Table S2), we see very similar phylogenies, but a significantly greater number of variants (*P* < 0.0001; χ^2^ with Yates correction) and elevation of TMRCA estimates for deep nodes (Supplemental Table S5), consistent with a reduction in missing data. Nonetheless, our MSY and mtDNA TMRCA estimates match well with independently published estimates (Supplemental Table S6), indicating that the loss of variants does not have a major effect on our overall conclusions.

### MSY sequence content across great ape lineages

Despite these caveats, our approach provides for the first time a broad picture of the sequence content of the X-degenerate (XDG) regions of MSY across great ape lineages. As noted above, the general reduction of recovered sequence is likely to be a consequence of the effect of sequence divergence upon capture efficiency. However, Figure 1B shows clearly the general retention of sequence content in the XDG regions, including the shared gametologous genes, while also highlighting some intergenic species-specific deletions and duplications with respect to the human reference. The only example of large-scale structural variation within species is seen in the Ptr2 structure found in the chimpanzees, Tommy and Moritz; we note that unusual cytogenetically detectable variation, including a pericentromeric inversion, has already been reported in Moritz (Schaller et al. 2010). Some MSY rearrangements are associated with reduced male fertility (Carvalho et al. 2011); although we do not have direct information on the fertility of Tommy and Moritz, the fact that they share the rearrangement and also a common paternal ancestor over 1000 generations ago, suggests that the Ptr2 structure is unlikely to have a deleterious effect on spermatogenesis. Deletion of short-arm-orthologous material in these individuals does remove two genes, *AMELY* and *TBL1Y*, which are adjacent in the chimpanzee assembly (Supplemental Fig. S5). Loss of these genes has also been documented in some human lineages (Jobling et al. 2007) and includes a recurrent event sponsored by nonallelic homologous recombination between *TSPY* repeats. The same mechanism cannot apply in chimpanzees, since the *TSPY* loci lie on the opposite arm of the Y Chromosome. Chimpanzee *TBL1Y* is described as a pseudogene (Perry et al. 2007; Bellott et al. 2014; Cortez et al. 2014), and although *AMELY* is apparently functional, the fact that its absence is tolerated, both within a long-lived chimpanzee lineage and in humans, supports the idea that it plays at most a minor functional role.

### MSY phylogenies and great ape subspecies relationships

Previously, a comparison has been made of a great ape mtDNA phylogeny with an autosomal tree based on a consensus of neighbor-joining trees for a large number of non-overlapping sequence blocks (Prado-Martinez et al. 2013). These two phylogenies showed high concordance, with monophyletic groupings for each species and subspecies. The MSY phylogenies produced here (Figs. 3, 4) agree with these analyses for all subspecies except two, eastern lowland gorillas and central chimpanzees. The gorilla GBG_Mkubwa is eastern lowland according to autosomal (Supplemental Fig. S3C) and mtDNA analysis (Fig. 3B), but is phylogenetically close to the mountain gorillas in the MSY tree, possibly indicating male-mediated gene flow among eastern gorillas. In the chimpanzees, autosomal and mtDNA analyses clearly support a split between central/eastern and western/Nigeria-Cameroon subspecies pairs. However, in our analysis, the deepest rooting branches in the MSY tree are found in central chimpanzees, which form a paraphyletic group, ancestral to the eastern chimpanzees, which are in turn ancestral to the sister clades of western and Nigeria-Cameroon subspecies (Fig. 3D). Of all four subspecies, central chimpanzees show the highest genome-wide nucleotide diversity (see Table 1) and effective population size (Prado-Martinez et al. 2013), so it is in this subspecies that ancient uniparentally inherited lineages are most likely to have survived, and disparities between mtDNA and MSY phylogenies are most likely to be observed.

### Dating nodes in the MSY phylogenies

TMRCA estimates are useful in allowing us to compare the depths of MSY and mtDNA phylogenies, accounting for differences in sequence lengths and mutation rates, although absolute values are uncertain. MSY-specific mutation rates for great apes are not available, so like others (Xue et al. 2015), we have used the published human rate, here based on the observation of 609 MSY mutations in Icelandic pedigrees (Helgason et al. 2015). A pedigree-based mutation study has been published based on chimpanzee autosomal sequences (Venn et al. 2014), and this yields an overall rate closely matching the human rate. However, the same study observes a higher male bias in mutation in chimpanzees than in humans, and this suggests that the human MSY mutation rate may actually underestimate the true chimpanzee rate. Clearly, more data on great ape mutation rates are needed.

If the human MSY mutation rate is a reasonable choice, it should lead to a reasonable estimate of the TMRCA of the human-chimpanzee divergence. The value we obtain is 6.91 (95% HPD interval: 6.11–7.79) MYA, which is not incompatible with the generally accepted divergence time of 6.5 MYA (Brunet et al. 2002; Vignaud et al. 2002), and suggests that use of the human rate is not wildly inappropriate.

### Inferences on dispersal and mating patterns

Here, we have compared MSY and mtDNA phylogenies in the same great ape individuals (Figs. 3, 4). It is worth emphasizing that there is no expectation that trees based on MSY and mtDNA should agree in their time depths, since each is an independent locus reflecting an independent realization of the evolutionary process. However, their sex-specific modes of inheritance mean that comparing the structures and time depths of phylogenies may provide information about sex-biased dispersal and mating patterns in great apes.

For orangutans, our sample sizes are too small to draw any reliable conclusions about sex bias. However, for gorillas, we observe low MSY diversity in both western and eastern species and consistently higher TMRCAs for mtDNA than for MSY, which is compatible with a polygynous mating system with dominant males in which drift acts strongly on MSY.

The chimpanzee MSY phylogeny contains remarkably deep-rooting nodes and has an overall TMRCA of 1148 (1011–1299) KYA; the mtDNA phylogeny has a similar overall TMRCA of 920 (811–1034) KYA. A multimale–multifemale mating system with female-biased dispersal might be expected to maintain high mtDNA diversity and also to facilitate the survival of MSY lineages, although these are likely to become geographically localized. Our data set is not suited to considering geographical localization within subspecies, but does allow a comparison among subspecies to be made. Here, we see striking differences: In the central, eastern, and Nigeria-Cameroon subspecies, both mtDNA and MSY show high diversity and the phylogenies contain deep-rooting nodes. However, western chimpanzees show a remarkably young TMRCA for MSY of 13.2 (10.8–15.8) KYA, combined with a value for mtDNA of 376 (312–444) KYA. Based on autosomal diversity, western chimpanzees have the smallest effective population size (5000) of the chimpanzee subspecies (Prado-Martinez et al. 2013), and this may have led to the loss of MSY lineages through drift. However, it is notable that our sample of four bonobo individuals, in which the autosomal-based species estimate of effective population size is also 5000 (Prado-Martinez et al. 2013), contains high MSY diversity with a TMRCA of 334 (294–379) KYA.

Sample sizes in our study are small, and this may affect the structures and time depths of phylogenies, and hence, the reliability of our conclusions. In principle, simulations could be used to investigate the influence of sampling effects, but require reasonable estimates for the parameters of reproductive success and migration rates between (sub)species that are currently unavailable. We have partially addressed this issue by comparing our TMRCA estimates with those from a number of independent studies of great ape MSY and mtDNA diversity (Supplemental Table S6). If our conclusions were strongly biased due to small sample sizes, and hence, missing key lineages, we might expect to see considerably older estimates in the literature compared to our results. However, in most cases, even when based upon significantly larger numbers of samples, literature estimates are of the same order as those that we report here. The exceptions are orangutans, which are known to possess considerable diversity in mtDNA (Nater et al. 2011) that we are missing in our very small sample.

### Influence of generation time on MSY species branch lengths

Our cross-species MSY phylogeny (Fig. 4) supports an apparent generation-time effect in species-specific branch lengths, in which lengths decrease in the order gorilla-chimpanzee-human, in parallel with published generation times (Fenner 2005; Langergraber et al. 2012). Generation time for bonobos is not recorded, but is likely to be similar to that of chimpanzees—the two species also show similar branch lengths. As we note above, human reference sequence bias in our study is likely to mean that the species differences in branch lengths here are underestimated.

A number of factors could contribute to lineage-specific effects on MSY branch lengths, including generation time, male-mutational bias, and paternal age effects. A study of the influence of life-history traits on phylogenetic base substitution rates in 32 mammalian genomes (Wilson Sayres et al. 2011) shows that generation time has the strongest effect, which is consistent with our findings.

### Future perspectives

The anthropocentric approach we have taken to great ape MSY diversity has yielded thousands of sequence variants and given a first view of the diverse structures and time depths of MSY phylogenies in our closest living relatives. Subspecies- and species-specific variants may prove useful in ape conservation, for example, in investigating the sources of illegally trafficked animals and bushmeat. Further improvement in our understanding of great ape population history and diversity will come from future developments, including accurate de novo MSY sequence assemblies from bonobos, gorillas, and orangutans, together with species-specific mutation rates and MSY data from larger sample sizes of geographically defined great apes.

## Methods

### DNA samples, sequencing, and data processing

Five-microgram aliquots of DNA from 19 great ape males (Supplemental Table S1) were used for library preparation and target enrichment (Agilent SureSelect) prior to sequencing on an Illumina HiSeq 2000 instrument with paired-end 100-bp run, at the Oxford Genomics Centre within the Wellcome Trust Centre for Human Genetics, University of Oxford, United Kingdom. All baits were designed based on the human reference sequence (GRCh37). Details of MSY bait design, coordinates, sequence data generation, and processing have been published previously (Hallast et al. 2015). For coordinates of autosomal and X-Chromosome regions analyzed here, see Supplemental Table S7.

Base calling was done using Illumina Bustard (Kao et al. 2009) and quality control with FastQC (http://www.bioinformatics.babraham.ac.uk/projects/fastqc/). Reads were mapped to the human genome reference (GRCh37) using Stampy v1.0.20 (Lunter and Goodson 2011). Remapping reads to the newer GRCh38 assembly is unlikely to alter our conclusions since the MSY sequence remains essentially unchanged between assemblies. Local realignment was done using The Genome Analysis Toolkit (GATK) v2.6-5 (DePristo et al. 2011), followed by duplicate read marking with Picard v1.86 (http://picard.sourceforge.net/) and base quality score recalibration with GATK. In order to determine if mapping to the human reference led to bias, we also mapped chimpanzee data to the chimpanzee genome reference (PanTro4) (see Supplemental Table S2; Supplemental Figs. S5, S7).

The mitochondrial genome (mtDNA) was amplified as two overlapping PCR fragments using published (Thalmann et al. 2004) primers (Cytbf, COIIrev592, 12So, and COII28for). Amplicons were pooled in equimolar amounts for each sample and barcoded. Sequence libraries were prepared using the NexteraXT kit (Illumina) according to the manufacturer's instructions and sequenced using Illumina MiSeq with 150-bp paired-end reads. Reads were mapped to the previously published mitochondrial assemblies of the corresponding species (chimpanzee: NC_001643.1; bonobo: NC_001644.1; gorilla: NC_011120.1; Sumatran orangutan: X97707.1; Bornean orangutan: NC_001646.1). Average coverage across samples was high (from 1200× to 1560×; mean 1460×). Data processing and variant calling were done as described above. For filters, see Supplemental Table S8, and for mitochondrial sequences, see Supplemental File S1.

All confident sites, single-nucleotide variants, and indels were called using the SAMtools (Li et al. 2009) mpileup v1.1 multisample option with the following general parameters: minimum base quality 20 and minimum mapping quality 30. Raw variants were filtered using VCFtools v0.1.12a (Danecek et al. 2011) and in-house Perl scripts (Supplemental File S2). Details and phylogenetic positions of all MSY variants shown in the trees in Figure 3 can be found by consulting Supplemental Figure S8 and Supplemental Tables S9–S13.

mtDNA sequences from all three data sets were aligned using Clustal Omega (Goujon et al. 2010; Sievers et al. 2011; McWilliam et al. 2013), and alignments were manually edited using AliView v1.17.1 (Larsson 2014). D-loop sequences were excluded from all analyses.

### Other data sets

Published data for multiple (sub)species (Prado-Martinez et al. 2013) were available as BAM files mapped to human genome reference hg18. All the variant calling and filtering steps used were identical to our data (see above). For filtered VCF files containing all confident sites, liftOver from hg18 to hg19 was done using Crossmap v0.1.5 software (http://crossmap.sourceforge.net/), followed by merging the overlapping sites with our filtered data set.

Data for mountain gorillas (Xue et al. 2015) were available as prefiltered VCF files mapped to human genome reference hg19 containing all confident sites. These were additionally filtered to match our data processing with no missing data allowed, followed by merging the overlapping sites from all data sets.

mtDNA data were available as FASTA files for both published data sets (Prado-Martinez et al. 2013; Xue et al. 2015).

To allow comparison with human data, we included eight MSY sequences picked to cover a wide variety of haplogroups; seven of these came from our previous study (Hallast et al. 2015), with the addition of a published A00 sequence (Karmin et al. 2015). Similarly, we included eight diverse whole mtDNA sequences: seven derived from the same sample set (C Batini, P Hallast, Å Vågene, D Zadik, MA Jobling, unpubl.) and one from a previously published study (Batini et al. 2011).

In order to obtain the orthologous MSY regions in all analyzed species, all samples were called simultaneously as described above. Among our samples (seven humans and 19 great apes), the total number of sites left after filtering was 1,269,652, and among male great apes (total of 21) from the large published data set (Prado-Martinez et al. 2013), it was 1,268,629. After merging the two, a total of 769,099 overlapping sites were left. Merging with the mountain gorilla data set (Xue et al. 2015) reduced this to 750,616 bp, including 54,611 variant sites. Therefore the length of orthologous MSY regions is somewhat longer than reported here, but sites were lost due to differences in the data sets (sequence capture versus whole-genome sequencing) and the strictness of filtering.

### Familial and ancestry analysis

Familial relationships among all analyzed great ape individuals were tested using the software KING (Manichaikul et al. 2010), and model-based analysis of ancestry was done using the program ADMIXTURE (Alexander et al. 2009); see Supplemental Text.

### Principal component analysis

Principal component analysis (PCA) was performed with the function “prcomp” in R environment version 3.0.2 (R Core Team 2014). For autosomal data sites with heterozygous calls in >80% of samples, triallelic sites and missing data were discarded to minimize background noise and uncertainty.

### Phylogenetic inference

PHYLIP v3.69 was used to create maximum parsimony phylogenetic trees (Felsenstein 2005) for both MSY and mtDNA. Three independent trees were constructed with DNAPARS using randomization of input order with different seeds, each 10 times. Output trees of these runs were used to build a consensus tree with the consense program included in the PHYLIP package.

Intraspecific MSY trees were rooted using the ancestral sequence generated and described in the Supplemental Text. Intraspecific mtDNA trees were rooted using the Human Revised Cambridge Reference Sequence (NC_012920.1). FigTree v1.4.0 (http://tree.bio.ed.ac.uk/software/figtree/) was used to visualize the tree.

### TMRCA and ages of nodes

The TMRCAs of nodes of interest were estimated using BEAST v1.8.1 (Drummond et al. 2005; Drummond and Rambaut 2007). In the absence of good estimates for great ape MSY mutation rates, we used the human rate of 3.07 (95% CI: 2.76–3.40) × 10^−8^ mutations/nucleotide/generation (Helgason et al. 2015). This was scaled according to the generation times (Langergraber et al. 2012) for each species (bonobos [assumed] and chimpanzees: 24 yr; gorillas and orangutans: 20 yr; humans: 30 yr) to mutations/nucleotide/year. For mtDNA, we considered only synonymous sites in the 13 protein-coding genes (nonsynonymous mutations were considered nonpolymorphic) and applied a human mutation rate of 1.113 × 10^−8^ mutations/nucleotide/year scaled for 11,395 bp (Soares et al. 2009). TMRCAs were also estimated (as done previously; Batini et al. 2011) based on an alternative rate of 1.1 (SE: 0.8–1.4) × 10^−8^ mutations/nucleotide/year estimated for the human coding region and scaled from the rate for the whole molecule using a coding/control region ratio of 1.57 (Supplemental Table S14; Soares et al. 2009). Markov chain Monte Carlo (MCMC) samples were based on 20,000,000 generations, logging every 1000 steps, with the first 2,000,000 generations discarded as burn-in. Three runs were combined for analysis using LogCombiner. We used a constant-sized coalescent tree prior and a strict clock. Substitution models to best fit the data were chosen according to the corrected Akaike Information Criterion (AICc) as implemented in MEGA5 (Tamura et al. 2011) and were as follows: HKY (humans, bonobos, gorillas, and orangutans) or GTR (chimpanzees) for MSY; and HKY+G (chimpanzees and gorillas), HKY (humans and bonobos), and TN93 (orangutans) for mtDNA. For MSY, only the variant sites were used and the number and composition of invariant sites was defined in the BEAST xml file. A prior with a normal distribution based on the 95% CI of the substitution rate was applied. TMRCAs were estimated in a single run, including all individuals per species and assigning samples to specific clades in agreement with the MP trees shown in Figure 3.

### Summary diversity statistics

Nucleotide diversity and its standard deviation were calculated using Arlequin v3.5.1.2 (Excoffier and Lischer 2010).

## Data access

Raw sequence data from this study have been submitted to the European Nucleotide Archive (ENA; http://www.ebi.ac.uk/ena/) under accession number PRJEB12247. Mitochondrial DNA sequences from this study have been submitted to Genbank (http://www.ncbi.nlm.nih.gov/genbank/) under accession numbers KU353708–KU353726 and are also in Supplemental File S1; MSY variant sites and genotypes in all samples and species are available in Supplemental Tables S9–S13. MSY variant sites from chimpanzees based on mapping to the chimpanzee genome reference (panTro4) have been submitted to dbSNP (http://www.ncbi.nlm.nih.gov/SNP/) under ss numbers given in Supplemental Table S13.

## Supplementary Material

Supplemental Material
