# Supplementary material for: Great ape Y Chromosome and mitochondrial DNA phylogenies reflect subspecies structure and patterns of mating and dispersal
Source: Genome Res. 2016 Apr;26(4):427–39. doi: 10.1101/gr.198754.115 (PMC4817767; doi:10.1101/gr.198754.115)
Supplement: Supplemental Material [file supp_26_4_427__index.html]

Great-ape Y Chromosome and mitochondrial DNA phylogenies reflect subspecies structure and patterns of mating and dispersal — Great ape Y Chromosome and mitochondrial DNA phylogenies reflect subspecies structure and patterns of mating and dispersal — Supplemental Material 

# Great ape Y Chromosome and mitochondrial DNA phylogenies reflect subspecies structure and patterns of mating and dispersal

## Supplemental Material

**Files in this Data Supplement:**

- Supp Material.pdf
- Supplemental FileS1\_mtDNA\_seq.txt
- Supplemental FileS2\_perl\_scripts.txt
- Supplemental TablesS7-S13.xlsx
