# Supplementary material for: Great ape Y Chromosome and mitochondrial DNA phylogenies reflect subspecies structure and patterns of mating and dispersal
Source: Genome Res. 2016 Apr;26(4):427–39. doi: 10.1101/gr.198754.115 (PMC4817767; doi:10.1101/gr.198754.115)
Supplement: Supplemental Material [file supp_gr.198754.115_SuppMaterial.pdf]

Supplemental Material

Supplemental Text

Analysis of chimpanzee data using the chimpanzee MSY reference sequence

Among the species analysed here, chimpanzee is the only one in addition to human for which a high-quality MSY reference sequence exists (Hughes et al. 2010). In order to check if using the human reference sequence (Skaletsky et al. 2003) biased the obtained results for MSY, we mapped all chimpanzee data to the panTro4 reference using Stampy v1.0.23 (Lunter and Goodson 2011). For samples from the published dataset (Prado-Martinez et al. 2013), reads were extracted from MSY BAM files mapped to the human genome reference using the Picard SamToFastq tool (Wysoker et al. 2009). All chimpanzee samples from both datasets were analysed together, and mapping and other data processing steps including filtering were done as described in the main text, except that GATK v3.4-0 (DePristo et al. 2011) was used for local realignment, and in variant calling minimum mapping quality 50 was used.

Variant calling for the chimpanzee MSY phylogeny was done on regions previously defined (Hughes et al. 2010) as X-degenerate and human-orthologous (panTro4 ChrY:16731094-21597282; ChrY:21647020-23798644; ChrY:24047549-25629393; ChrY:25794387-26265354), totalling approximately 9 Mb, with mean coverage ranging from 17 × to 26.6 × (mean 22 ×) for our samples, and from 6.4 × to 18.6 × (mean 10.6 ×) for published (Prado-Martinez et al. 2013) samples. A total of 2,336,730 bp, including 12,127 variant sites, were left after filtering from the total of 19 chimpanzees.

Based on the chimpanzee reference sequence analysis we constructed a maximum parsimony tree (Supplemental Figure S7) and also estimated TMRCAs of the nodes labelled in the tree shown in Figure 3, obtaining very similar dates (Supplemental Table S5).

Comparison of custom-capture and published whole-genome sequence (WGS) datasets

A comparison of MSY sequence data obtained using custom enrichment and whole-genome sequencing was undertaken to investigate possible biases created by the capture approach. The capture probes were built on the human reference genome, therefore possibly enriching for regions of highest homology between great apes and humans. This is expected to affect the MSY regions the most, as autosomes and X Chromosome have diverged less over evolutionary time (Hughes 2010).

We analysed separately the capture and WGS data mapped to the human genome reference sequence for bonobos, chimpanzees, gorillas and orangutans using identical variant calling and filtering parameters, as described for the final merged MSY dataset. This procedure leads to somewhat different final lengths of sequence between the two sequencing approaches (Supplemental Table S3), mainly due to differences in read-depth, and also a further reduced length when data from both

approaches are merged into an overlapping set. We observe statistically significant differences between WGS and sequence-capture in the mean number of variant sites in each species. However, no significant differences are seen between capture and WGS samples for any of the species when these are extracted from the final merged dataset (Supplemental Table S3). Estimates of divergence from human for individual samples, whether based on sequence-capture, WGS, or the merged dataset, are very similar (Supplemental Table S4), showing that when overlapping MSY regions are retained then the datasets behave in the same way.

The obtained differences between capture and WGS data are likely due to a combination of factors such as inappropriate reference sequence, differences in read lengths and depth of coverage, and in the specific MSY regions retained in each analysis. Different regions of a chromosome may differ in mutation rate, as has been reported recently for MSY (Trombetta et al. 2015). The reference sequence effect can neither be overcome nor properly evaluated until high-quality MSY sequences for all species become available. Notably, when mapping chimpanzees to the chimpanzee reference, we see an increase in the total number of variants identified, but the fact that TMRCA estimates are only very slightly affected suggests a minor effect on the overall results and conclusions of our analysis.

Concordance of genotype calls

Overlap of some great-ape samples between datasets allowed an assessment of genotype concordance and therefore also gave an indication of data quality in our sequencing.

For mtDNA, GGG_Guy was sequenced both by us and within a published dataset (Xue et al. 2015), and PPA_Bono was sequenced by us and in a published dataset (Prado-Martinez et al. 2013). In each case, comparing respectively 15,453 and 15,447 bp, there were no discordant calls.

For MSY, PPA_Bono was sequenced by us and in a published dataset (Prado-Martinez et al. 2013), and showed no discordant calls out of the 54,695 variant sites used to build the cross-species phylogeny shown in Figure 4.

For autosomal data a total of 28 genotypes out of 14,024 variant sites differed for PPA_Bono between the datasets, suggesting an overall discordance rate ~0.2%.

Relatedness analysis

Relatedness is known to be a problem when using great-ape samples, in particular for captive-born individuals. For our samples we had no prior knowledge of possible relatedness, except for gorillas GGG_Nikumba and GGG_Tomoka, who have been reported to be father and son (Boyer et al. 1973). Also, some individuals were known to be closely related based on published genetic data (Prado-Martinez et al. 2013; Xue et al. 2015). In order to estimate the kinship coefficients between all samples, the software KING was used (Manichaikul et al. 2010). Among bonobos, no relatedness was identified. Among chimpanzees, a second-degree relationship was

identified between PTV_8 and PTV_Donald, but since their MSY sequences differ by 82 variants across the final 2,496,576 bp, both samples were retained in all MSY analyses. However, because no differences were found in the mtDNAs between the two, PTV_Donald was retained in the mtDNA tree, but removed from dating and calculations of genetic diversity. Among gorillas, the father-son relationship between GGG_Nikumba and GGG_Tomoka was confirmed. For other gorillas we obtained the same relationships as previously published (Prado-Martinez et al. 2013; Xue et al. 2015). Among orangutans, first-degree relationships were found between PPY_Thai and PPY_Temmy, and PAB_Sinjo and PAB_Babu.

ADMIXTURE analysis

Analysis of shared genetic components in autosomal data was done using the ADMIXTURE program (Alexander et al. 2009) with 10-fold cross-validation (CV) (Alexander and Lange 2011). In order to avoid biases from related individuals, the following samples were excluded: PTV_Donald, GGG_Tomoka, GGG_Bulera, GGG_Suzie, GGG_Oko, GGG_Kowali, GBB_Umurimo, GBB_Maisha, PPY_Temmy and PAB_Babu. Overall the results (Supplemental Figure S3) agree well with the PCAs (Figure 2; Supplemental Figure S1). The CV error is lowest for k=1 for bonobos, k=3 for chimpanzees, and k=2 for gorillas and orangutans (Supplemental Figure S4). For the final number of autosomal variants used for each species, see Supplemental Table S2.

Ancestral states of MSY variants

In order to define the ancestral states for the final filtered variants used in intraspecific phylogenetic tree construction (Figure 3), we used sequence data generated from the other species (including humans), as well as the phylogenetic information from the maximum parsimony tree extracted from the PHYLIP outfile.

First, final variant sites from the species to be rooted were called from other species using SAMTools (Li et al. 2009) as described above, filtered for strand bias and minimum depth $\geq$ 1. For rooting the bonobo and chimpanzee trees, the allele matching gorillas or orangutans was preferred, and when missing data or non-matching alleles precluded this, the human allele was used. For gorillas, the orangutan allele was preferred and for orangutans the gorilla allele was preferred. If the sites were missing in the other species or alleles did not match, then the ancestral allele was assigned randomly.

The allelic state defined using other species was checked against the reconstructed ancestral state from the PHYLIP output. If the defined allele did not match with the deepest rooting branches, but an allele from a different species did, then the ancestral state was changed and after checking all sites the maximum parsimony tree was constructed again. For all species this left a small proportion of sites (0.12-1.82% of variants) where the ancestral state was not supported by reconstructed states in the PHYLIP output.

Estimation of human-chimpanzee MSY divergence time based on sequence data

To ask if using the human MSY mutation rate was reasonable, we used it to estimate the divergence time of humans and chimpanzees. Human and chimpanzee samples were called and filtered together, yielding a total of 43,752 variants across 2,171,604 bp. Generation time differences were accounted for by averaging the per-nucleotide-per-year mutation rates. We used a Yule process speciation tree prior, a strict clock with normal distribution and HKY substitution model.

Supplemental References

Alexander DH, Lange K. 2011. Enhancements to the ADMIXTURE algorithm for individual ancestry estimation. *BMC bioinformatics* **12**: 246.

Alexander DH, Novembre J, Lange K. 2009. Fast model-based estimation of ancestry in unrelated individuals. *Genome Res* **19**: 1655-1664.

Arora N, Nater A, van Schaik CP, Willems EP, van Noordwijk MA, Goossens B, Morf N, Bastian M, Knott C, Morrogh-Bernard H et al. 2010. Effects of Pleistocene glaciations and rivers on the population structure of Bornean orangutans (Pongo pygmaeus). *Proc Natl Acad Sci U S A* **107**: 21376-21381.

Batini C, Lopes J, Behar DM, Calafell F, Jorde LB, van der Veen L, Quintana-Murci L, Spedini G, Destro-Bisol G, Comas D. 2011. Insights into the demographic history of African Pygmies from complete mitochondrial genomes. *Mol Biol Evol* **28**: 1099-1110.

Bjork A, Liu W, Wertheim JO, Hahn BH, Worobey M. 2011. Evolutionary history of chimpanzees inferred from complete mitochondrial genomes. *Mol Biol Evol* **28**: 615-623.

Boyer SH, Noyes AN, Boyer ML, Marr K. 1973. Hemoglobin alpha-3 chains in apes. Primary structures and the presumptive nature of back mutation in a normally silent gene. *J Biol Chem* **248**: 992-1003.

Das R, Hergenrother SD, Soto-Calderon ID, Dew JL, Anthony NM, Jensen-Seaman MI. 2014. Complete mitochondrial genome sequence of the Eastern gorilla (Gorilla beringei) and implications for african ape biogeography. *J Hered* **105**: 752-761.

DePristo MA, Banks E, Poplin R, Garimella KV, Maguire JR, Hartl C, Philippakis AA, del Angel G, Rivas MA, Hanna M et al. 2011. A framework for variation discovery and genotyping using next-generation DNA sequencing data. *Nat Genet* **43**: 491-498.

Eriksson J, Siedel H, Lukas D, Kayser M, Erler A, Hashimoto C, Hohmann G, Boesch C, Vigilant L. 2006. Y-chromosome analysis confirms highly sex-biased dispersal and suggests a low male effective population size in bonobos (Pan paniscus). *Mol Ecol* **15**: 939-949.

Hallast P, Batini C, Zadik D, Maisano Delser P, Wetton JH, Arroyo-Pardo E, Cavalleri GL, de Knijff P, Destro Bisol G, Dupuy BM et al. 2015. The Y-chromosome tree bursts into leaf: 13,000 high-confidence SNPs covering the majority of known clades. *Mol Biol Evol* **32**: 661-673.

Hughes JF, Skaletsky H, Pyntikova T, Graves TA, van Daalen SK, Minx PJ, Fulton RS, McGrath SD, Locke DP, Friedman C et al. 2010. Chimpanzee and human

Y chromosomes are remarkably divergent in structure and gene content. *Nature* **463**: 536-539.
Hvilsom C, Carlsen F, Heller R, Jaffre N, Siegismund HR. 2014. Contrasting demographic histories of the neighboring bonobo and chimpanzee. *Primates* **55**: 101-112.
Karmin M, Saag L, Vicente M, Wilson Sayres MA, Jarve M, Talas UG, Rootsi S, Ilumae AM, Magi R, Mitt M et al. 2015. A recent bottleneck of Y chromosome diversity coincides with a global change in culture. *Genome Res* **25**: 459-466.
Li H, Handsaker B, Wysoker A, Fennell T, Ruan J, Homer N, Marth G, Abecasis G, Durbin R, Genome Project Data Processing S. 2009. The Sequence Alignment/Map format and SAMtools. *Bioinformatics* **25**: 2078-2079.
Lunter G, Goodson M. 2011. Stampy: a statistical algorithm for sensitive and fast mapping of Illumina sequence reads. *Genome Res* **21**: 936-939.
Ma X, Kelley JL, Eilertson K, Musharoff S, Degenhardt JD, Martins AL, Vinar T, Kosiol C, Siepel A, Gutenkunst RN et al. 2013. Population genomic analysis reveals a rich speciation and demographic history of orang-utans (Pongo pygmaeus and Pongo abelii). *PLoS One* **8**: e77175.
Manichaikul A, Mychaleckyj JC, Rich SS, Daly K, Sale M, Chen WM. 2010. Robust relationship inference in genome-wide association studies. *Bioinformatics* **26**: 2867-2873.
Nater A, Nietlisbach P, Arora N, van Schaik CP, van Noordwijk MA, Willems EP, Singleton I, Wich SA, Goossens B, Warren KS et al. 2011. Sex-biased dispersal and volcanic activities shaped phylogeographic patterns of extant Orangutans (genus: Pongo). *Mol Biol Evol* **28**: 2275-2288.
Oetjens MT, Shen F, Zou Z, Kidd JM. 2015. Inferring chimpanzee Y chromosome history and amplicon diversity from whole genome sequencing. *BioRxiv* doi: org/10.1101/029702.
Prado-Martinez J, Sudmant PH, Kidd JM, Li H, Kelley JL, Lorente-Galdos B, Veeramah KR, Woerner AE, O'Connor TD, Santpere G et al. 2013. Great ape genetic diversity and population history. *Nature* **499**: 471-475.
Skaletsky H, Kuroda-Kawaguchi T, Minx PJ, Cordum HS, Hillier L, Brown LG, Repping S, Pyntikova R, Ali J, Bieri T et al. 2003. The male-specific region of the human Y chromosome: a mosaic of discrete sequence classes. *Nature* **423**: 825-837.
Stone AC, Griffiths RC, Zegura SL, Hammer MF. 2002. High levels of Y-chromosome nucleotide diversity in the genus *Pan*. *Proc Natl Acad Sci U S A* **99**: 43-48.
Stone AC, Battistuzzi FU, Kubatko LS, Perry GH, Jr., Trudeau E, Lin H, Kumar S. 2010. More reliable estimates of divergence times in Pan using complete mtDNA sequences and accounting for population structure. *Philosophical Transactions of the Royal Society of London Series B, Biological sciences* **365**: 3277-3288.
Trombetta B, D'Atanasio E, Massaia A, Myres NM, Scozzari R, Cruciani F, Novelletto A. 2015. Regional differences in the accumulation of SNPs on the male-specific portion of the human Y chromosome replicate autosomal patterns: implications for genetic dating. *PLoS One* **10**: e0134646.
Wysoker A, Tibbetts K, Fennell T. 2009. Picard v1.86, available from http://picard.sourceforge.net/.

Xue Y, Prado-Martinez J, Sudmant PH, Narasimhan V, Ayub Q, Szpak M, Frandsen P, Chen Y, Yngvadottir B, Cooper DN et al. 2015. Mountain gorilla genomes reveal the impact of long-term population decline and inbreeding. *Science* **348**: 242-245.

Zsurka G, Kudina T, Peeva V, Hallmann K, Elger CE, Khrapko K, Kunz WS. 2010. Distinct patterns of mitochondrial genome diversity in bonobos (Pan paniscus) and humans. *BMC Evol Biol* **10**: 270.

Supplemental Table S1: Great-ape and human individuals sequenced and analysed in this study.

Species	Common name	Name	Name in figures	Sex	Studbook ID	Geographic origin/ population/birth origin	MSY/mtDNA lineage	Ref
Pan paniscus	Bonobo	Bono	PPA_Bono	M	102	Wild born	na	
P. paniscus	Bonobo	PPA2	PPA_PPA2	M	na	Captive born	na	
P. paniscus	Bonobo	Masikini	PPA_Masikini	M	54	Wild born	na	
Pan troglodytes troglodytes	Central chimpanzee	Moritz	PTT_Moritz	M	10920	Nigeria, wild born	na	
P. t. troglodytes/ Pan troglodytes verus	hybrid	Tommy	PTT/PTV_Tommy	M	na	Wild born?	na	
P. t. schweinfurthii	Eastern chimpanzee	Bobby	PTS_Bobby	M	na	Wild born?	na	
P. t. verus	Western chimpanzee	PTR7 - Johnny	PTV_7	M	na	Captive born	na	
P. t. verus	Western chimpanzee	Buttons	PTV_Buttons	M	10411		na	
P. t. verus	Western chimpanzee	NA03450	PTV_NA03450	M	na	Coriell Institute for Medical Research	na	
P. t. verus	Western chimpanzee	PTR8 - Carl	PTV_8	M	na	Captive born	na	
P. t. verus/ P. t. ellioti	hybrid	EB176JC	PTE/PTV_EB176JC	M	na	ECACC cell line no. 89072704	na	
Gorilla gorilla gorilla	Western lowland gorilla	Fritz	GGG_Fritz	M	253	Cameroon, wild born	na	
G. gorilla gorilla	Western lowland gorilla	Tomoka	GGG_Tomoka	M	122	Captive born	na	
G. g. gorilla	Western lowland gorilla	Guy	GGG_Guy	M	5	Cameroon, wild born	na	
G. g. gorilla	Western lowland gorilla	Nikumba	GGG_Nikumba	M	42	Republic of the Congo (?), wild born	na	
Pongo abelii	Sumatran orangutan	Sinjo	PAB_Sinjo	M	1007	Captive born	na	
P. abelii	Sumatran orangutan	Duapuluh	PAB_Duapuluh	M	2810	Captive born	na	
P. abelii	Sumatran orangutan	ppy10	PAB_ppy10	M	na	Captive born	na	
P. pygmaeus	Bornean orangutan	Thai	PPY_Thai	M	2861	Captive born	na	
**Human samples used for MSY**								
Homo sapiens	Human	GRC13292546	HSA_A00	M		Mbo	A00	1
H. sapiens	Human	bkl-2	HSA_A0	M		Bakola	A0-L896	2

H. sapiens	Human	eng-GB1778	HSA_A1a	M		English	A1a-M31	2
H. sapiens	Human	bak-41	HSA_A2	M		Baka	A2-M14	2
H. sapiens	Human	bak-25	HSA_B2a	M		Baka	B2a1a-M152	2
H. sapiens	Human	nep-0273	HSA_C5	M		Nepalese	C5-M365	2
H. sapiens	Human	bav-13	HSA_E1b	M		Bavarian	E1b1b1c-M123	2
H. sapiens	Human	ire-55	HSA_R1b	M		Irish	R1b1b2-M222	2
**Human samples used for mtDNA**								
H. sapiens	Human	eng-O109	HSA_H	M		English	H	2
H. sapiens	Human	CHB-NA18636	HSA_M	M		Chinese (CHB)	M	2
H. sapiens	Human	CHB-NA18611	HSA_A	M		Chinese (CHB)	A	2
H. sapiens	Human	CHB-NA18562	HSA_D	M		Chinese (CHB)	D	2
H. sapiens	Human	ork-565	HSA_U4	M		Orcadian	U4	2
H. sapiens	Human	YRI-NA18501	HSA_L1	M		Yoruba (YRI)	L1	2
H. sapiens	Human	YRI-NA18856	HSA_L2	M		Yoruba (YRI)	L2	2
H. sapiens	Human	H079	HSA_L0	M		Mbuti	L0a	3
**Great ape samples from published datasets**								
P. paniscus	Bonobo	Desmond	PPA_Desmond	M	57	Wild born	na	4
P. paniscus	Bonobo	Bono	PPA_Bono	M	102	Wild born	na	4
P. paniscus	Bonobo	LB502	PPA_LB502	F	LB502	Captive born	na	4
P. paniscus	Bonobo	Hortense	PPA_Hortense	F	91	Wild born	na	4
P. paniscus	Bonobo	Kosana	PPA_Kosana	F	166	Wild born	na	4
P. paniscus	Bonobo	Dzeeta	PPA_Dzeeta	F	67	Wild born	na	4
P. paniscus	Bonobo	Hermien	PPA_Hermien	F	88	Wild born	na	4

P. paniscus	Bonobo	Catherine	PPA_Catherine	F	55	Wild born	na	4
P. paniscus	Bonobo	Kombote	PPA_Kombote	F	56	Wild born	na	4
P. paniscus	Bonobo	Chipita	PPA_Chipita	F	220	Wild born	na	4
P. paniscus	Bonobo	Natalie	PPA_Natalie	F	46	Wild born	na	4
P. paniscus	Bonobo	Salonga	PPA_Salonga	F	52	Captive born	na	4
P. paniscus	Bonobo	Kumbuka	PPA_Kumbuka	F	260	Captive born	na	4
P. t. troglodytes	Central chimpanzee	Vaillant	PTT_Vaillant	M	na	Wild born	na	4
P. t. troglodytes	Central chimpanzee	Doris	PTT_Doris	F	na	Wild born	na	4
P. t. troglodytes	Central chimpanzee	Julie	PTT_Julie	F	10306?	Wild born	na	4
P. t. troglodytes	Central chimpanzee	Clara	PTT_Clara	F	na	Wild born	na	4
P. t. schweinfurthii	Eastern chimpanzee	Vincent	PTS_Vincent	M	Ch-045	Wild born	na	4
P. t. schweinfurthii	Eastern chimpanzee	Bwambale	PTS_Bwambale	M	N/A	Wild born	na	4
P. t. schweinfurthii	Eastern chimpanzee	Andromeda	PTS_Andromeda	F	BB-042a	Wild born	na	4
P. t. schweinfurthii	Eastern chimpanzee	Harriet	PTS_Harriet	F	ISIS #925	Wild born	na	4
P. t. schweinfurthii	Eastern chimpanzee	Kidongo	PTS_Kidongo	F	N/A	Wild born	na	4
P. t. schweinfurthii	Eastern chimpanzee	Nakuu	PTS_Nakuu	F	N/A	Wild born	na	4
P. t. verus	Western chimpanzee	Bosco	PTV_Bosco	M	215?	Wild born	na	4
P. t. verus	Western chimpanzee	Koby	PTV_Koby	M	650?	Wild born	na	4
P. t. verus	Western chimpanzee	Clint	PTV_Clint	M	C0471	Captive born	na	4
P. t. verus/ P. t. troglodytes	hybrid	Donald	PTV_Donald	M	C0551	Captive born	na	4
P. t. verus	Western chimpanzee	Jimmie	PTV_Jimmie	F	10784	Wild born	na	4
P. t. ellioti	Nigeria-Cameroon chimpanzee	Akwaya-Jean	PTE_Akwaya-Jean	M	LWC2	Wild born	na	4
P. t. ellioti	Nigeria- Cameroon chimpanzee	Basho	PTE_Basho	M	LWC8	Wild born	na	4
P. t. ellioti	Nigeria-Cameroon chimpanzee	Damian	PTE_Damian	M	LWC12	Wild born	na	4
P. t. ellioti	Nigeria-Cameroon chimpanzee	Koto	PTE_Koto	M	LWC24	Wild born	na	4
P. t. ellioti	Nigeria-Cameroon chimpanzee	Taweh*	PTE_Taweh	M	LWC43	Wild born	na	4
P. t. ellioti	Nigeria-Cameroon chimpanzee	Banyo	PTE_Banyo	F	LWC7	Wild born	na	4

P. t. ellioti	Nigeria-Cameroon chimpanzee	Julie	PTE_Julie	F	LWC21	Wild born	na	4
P. t. ellioti	Nigeria-Cameroon chimpanzee	Kopongo	PTE_Kopongo	F	LWC23	Wild born	na	4
P. t. ellioti	Nigeria-Cameroon chimpanzee	Paquita	PTE_Paquita	F	LWC038	Wild born	na	4
P. t. ellioti	Nigeria-Cameroon chimpanzee	Tobi	PTE_Tobi	F	LWC046	Wild born	na	4
G. beringei graueri	Eastern lowland gorilla	M'kubwa	GBG_Mkubwa	M	9907	Wild born	na	4
G. b. graueri	Eastern lowland gorilla	Kaisi	GBG_Kaisi	M	9909	Wild born	na	4
G. b. graueri	Eastern lowland gorilla	Ntabwoba	GBG_Ntabwoba	M	na	Wild born	na	5
G. b. graueri	Eastern lowland gorilla	Victoria	GBG_Victoria	F	9919	Captive born	na	4
G. b. graueri	Eastern lowland gorilla	Dunia	GBG_Dunia	F	na	Wild born	na	5
G. b. graueri	Eastern lowland gorilla	Itebero	GBG_Itebero	F	na	Wild born	na	5
G. b. graueri	Eastern lowland gorilla	Pinga	GBG_Pinga	F	na	Wild born	na	5
G. b. graueri	Eastern lowland gorilla	Serufuli	GBG_Serufuli	F	na	Wild born	na	5
G. b. graueri	Eastern lowland gorilla	Tumani	GBG_Tumani	F	na	Wild born	na	5
G. b. beringei	Mountain gorilla	Imfura	GBB_Imfura	M	na	Wild born	na	5
G. b. beringei	Mountain gorilla	Kaboko	GBB_Kaboko	M	na	Wild born	na	5
G. b. beringei	Mountain gorilla	Zirikana	GBB_Zirikana	M	na	Wild born	na	5
G. b. beringei	Mountain gorilla	Maisha	BGG_Maisha	F	na	Wild born	na	5
G. b. beringei	Mountain gorilla	Tuck	GBB_Tuck	F	na	Wild born	na	5
G. b. beringei	Mountain gorilla	Turimaso	GBB_Turimaso	F	na	Wild born	na	5
G. b. beringei	Mountain gorilla	Umurimo	GBB_Umurimo	F	na	Wild born	na	5
G. g. gorilla	Western lowland gorilla	Azizi	GGG_Azizi	M	1459	Captive born	na	4
G. g. gorilla	Western lowland gorilla	Banjo	GGG_Banjo	M	255	Wild born	na	4
G. g. gorilla	Western lowland gorilla	Abe	GGG_Abe	M	52	Wild born	na	4
G. g. gorilla	Western lowland gorilla	Tzambo	GGG_Tzambo	M	440	Wild born	na	4
G. g. gorilla	Western lowland gorilla	Kowali	GGG_Kowali	F	663	Captive born	na	4
G. g. gorilla	Western lowland gorilla	Bulera	GGG_Bulera	F	1120	Captive born	na	4
G. g. gorilla	Western lowland gorilla	Suzie	GGG_Suzie	F	636	Wild born	na	4
G. g. gorilla	Western lowland gorilla	Kokamo	GGG_Kokamo	F	1049	Captive born	na	4
G. g. gorilla	Western lowland gorilla	Mimi	GGG_Mimi	F	241	Wild born	na	4
G. g. gorilla	Western lowland gorilla	Dian	GGG_Dian	F	1091	Captive born	na	4

G. g. gorilla	Western lowland gorilla	Delphi	GGG_Delphi	F	230	Wild born	na	4
G. g. gorilla	Western lowland gorilla	Sandra	GGG_Sandra	F	969	Captive born	na	4
G. g. gorilla	Western lowland gorilla	Coco	GGG_Coco	F	1351	Wild born	na	4
G. g. gorilla	Western lowland gorilla	Kolo	GGG_Kolo	F	936	Captive born	na	4
G. g. gorilla	Western lowland gorilla	Amani	GGG_Amani	F	899	Captive born	na	4
G. g. gorilla	Western lowland gorilla	Akiba Beri	GGG_Akiba_Beri	F	1926	Wild born	na	4
G. g. gorilla	Western lowland gorilla	Choomba	GGG_Choomba	F	180	Wild born	na	4
G. g. gorilla	Western lowland gorilla	Paki	GGG_Paki	F	191	Wild born	na	4
G. g. gorilla	Western lowland gorilla	Anthal	GGG_Anthal	F	1930	Wild born	na	4
G. g. gorilla	Western lowland gorilla	Katie	GGG-B650_Katie	F	498	Wild born	na	4
G. g. gorilla	Western lowland gorilla	Carolyn	GGG_Carolyn	F	3	Wild born	na	4
G. g. gorilla	Western lowland gorilla	Porta	GGG_Porta	F	64	Wild born	na	4
G. g. gorilla	Western lowland gorilla	Vila	GGG_Vila	F	80	Wild born	na	4
G. g. gorilla	Western lowland gorilla	Helen	GGG_Helen	F	96	Wild born	na	4
G. g. gorilla	Western lowland gorilla	Oko	GGG_Oko	F	192	Wild born	na	4
G. g. gorilla	Western lowland gorilla	Dolly	GGG_Dolly	F	195	Wild born	na	4
G. g. gorilla	Western lowland gorilla	Katie	GGG-KB4986_Katie	F	498	Wild born	na	4
G. g. diehli	Cross River gorilla	Nyango	GGD_Nyango	F	9941	Wild born	na	4
P. abelii	Sumatran orangutan	Buschi	PAB_Buschi	M	498	Wild born	na	4
P. abelii	Sumatran orangutan	Elsi	PAB_Elsi	F	446	Wild born	na	4
P. abelii	Sumatran orangutan	Kiki	PAB_Kiki	F	154	Wild born	na	4
P. abelii	Sumatran orangutan	Dunja	PAB_Dunja	F	1302	Captive born	na	4
P. abelii	Sumatran orangutan	Babu	PAB_Babu	F	511	Wild born	na	4
P. pygmaeus	Bornean orangutan	Napoleon	PPY_Napoleon	M	898	Wild born	na	4
P. pygmaeus	Bornean orangutan	Nonja	PPY_Nonja	F	1581	Captive born	na	4
P. pygmaeus	Bornean orangutan	Temmy	PPY_Temmy	F	1852	Captive born	na	4
P. pygmaeus	Bornean orangutan	Sari	PPY_Sari	F	1097	Captive born	na	4
P. pygmaeus	Bornean orangutan	Tilda	PPY_Tilda	F	1452	Wild born	na	4

Refs.: 1 – Karmin et al. 2015; 2 – Hallast et al. 2015; 3 - Batini et al. 2011; 4 - Prado-Martinez et al. 2013; 5 - Xue et al. 2015
* - according to Prado-Martinez et al. 2013, Taweh seems to have two X Chromosomes plus a portion of the Y Chromosome, therefore in this study only autosomal regions were used from this sample; na – not available.

Supplemental Table S2: Summary of variants and sequenced regions.

	N	Final length	S	Recurrent sites (%)
**MSY (hg19)**				
All samples	51	750,616	54,611	na
Humans	8	3,587,037	3544	na
Chimpanzees	19	2,496,576	12,208	68 (0.56%)
Bonobos	4	3,637,523	3284	0 (0%)
Chimpanzees and bonobos	23	2,340,169	18,753	na
Gorillas	13	2,043,299	1262	4 (0.32%)
Orangutans	6	2,348,840	2476	8 (0.32%)
**MSY (PanTro4)**				
Chimpanzees	19	2,336,730	12,127	87 (0.72%)
**ChrX**				
Chimpanzees	19	211,364	1070	na
Bonobos	4	268,948	220	na
Gorillas	14	72,367	171	na
Orangutans	6	247,819	890	na
**Autosomal**				
Chimpanzees	33	3,666,900	27,376	na
Bonobos	15	5,092,536	13,996	na
Gorillas	48	2,627,902	10,069	na
Orangutans	14	5,364,020	48,109	na

N - sample size; S – number of variable sites; na – not available.

Supplemental Table S3: Comparison of MSY data based on custom-capture and whole-genome sequencing.

Dataset*	N	Final length	Mean no. of REF sites	Mean no. of ALT sites	Mean divergence from human	p-value**
**Bonobos**						
WGS	2	3,994,211	3,933,780	60,431	1.513	0.003 (WGS vs capt.)
capture	3	3,799,793	3,743,280	56,513	1.487	0.406 (capt. vs merged)
merged	4	3,637,523	3,583,692	53,831	1.480	<0.0001 (WGS vs merged)
merged-WGS	2	3,637,523	3,583,681	53,843	1.480	0.949 (merged-WGS vs merged-capt.)
merged-capture	3	3,637,523	3,583,702	53,821	1.479	
**Chimpanzees**						
WGS	11	3,075,888	3,032,804	43,084	1.401	<0.0001 (WGS vs capt.)
capture	8	3,297,041	3,247,697	49,344	1.497	<0.0001 (capt. vs merged)
merged	19	2,496,576	2,460,684	35,892	1.438	<0.0001 (WGS vs merged)
merged-WGS	11	2,496,576	2,460,712	35,864	1.436	0.804 (merged-WGS vs merged-capt.)
merged-capture	8	2,496,576	2,460,645	35,931	1.439	
**Gorillas**						
WGS	10	2,071,756	2,031,405	40,351	1.948	<0.0001 (WGS vs capt.)
capture	4	2,789,895	2,732,957	56,938	2.041	<0.0001 (capt. vs merged)
merged	14	2,043,299	2,003,432	39,867	1.951	0.803 (WGS vs merged)
merged-WGS	10	2,043,299	2,003,437	39,862	1.951	0.957 (merged-WGS vs merged-capt.)
merged-capture	4	2,043,299	2,003,421	39,878	1.952	
**Orangutans**						
WGS	2	2,850,916	2,709,102	141,815	4.974	<0.0001 (WGS vs capt.)
capture	4	2,537,735	2,426,470	111,266	4.384	0.018 (capt. vs merged)
merged	6	2,348,840	2,246,887	101,954	4.341	<0.0001 (WGS vs merged)
merged-WGS	2	2,348,840	2,246,865	101,975	4.341	0.944 (merged-WGS vs merged-capt.)
merged-capture	4	2,348,840	2,246,897	101,943	4.340	

* - WGS - whole-genome sequenced samples; capture - samples sequenced using custom-capture approach (current study); merged - WGS and capture samples analysed together to obtain overlapping sites that were used in all analysis undertaken; merged-WGS/merged-capture - WGS/capture samples extracted from the final merged dataset
** - chi square with Yates correction

Supplemental Table S4: Comparison of individual sample data based on custom-capture and whole-genome sequencing.

Sample	Dataset	Final length	Fixed differen ces	Variant sites (REF calls)	Varian t sites (ALT calls)	Diver-gence from human	Dataset	Final length	Fixed differen ces	Varian t sites (REF calls)	Varian t sites (ALT calls)	Diver-gence from human
**Bonobos**												
PPA_Desmond	WGS	3,994,211	58,668	1706	1767	1.513	merged-WGS	3,637,523	52,259	1684	1600	1.481
PPA_Bono	WGS	3,994,211	58,668	1714	1759	1.513	merged-WGS	3,637,523	52,259	1717	1567	1.480
PPA_Bono	capture	3,799,793	56,323	186	196	1.487	merged-capt.	3,637,523	52,259	1717	1567	1.480
PPA_PPA2	capture	3,799,793	56,323	194	188	1.487	merged-capt.	3,637,523	52,259	1725	1559	1.480
PPA_Masikini	capture	3,799,793	56,323	197	185	1.487	merged-capt.	3,637,523	52,259	1723	1561	1.480
**Chimpanzees**												
PTE_Akwaya_Jean	WGS	3,075,888	41,113	5420	2065	1.404	merged-WGS	2,496,576	32,086	8380	3828	1.439
PTE_Basho	WGS	3,075,888	41,113	5575	1910	1.399	merged-WGS	2,496,576	32,086	8511	3697	1.433
PTE_Damian	WGS	3,075,888	41,113	5426	2059	1.404	merged-WGS	2,496,576	32,086	8383	3825	1.438
PTE_Koto	WGS	3,075,888	41,113	5421	2064	1.404	merged-WGS	2,496,576	32,086	8380	3828	1.439
PTS_Vincent	WGS	3,075,888	41,113	5563	1922	1.399	merged-WGS	2,496,576	32,086	8448	3760	1.436
PTS_Bwambale	WGS	3,075,888	41,113	5608	1877	1.398	merged-WGS	2,496,576	32,086	8487	3721	1.434
PTT_Vaillant	WGS	3,075,888	41,113	5546	1939	1.400	merged-WGS	2,496,576	32,086	8495	3713	1.434
PTV_Bosco	WGS	3,075,888	41,113	5526	1959	1.400	merged-WGS	2,496,576	32,086	8414	3794	1.437
PTV_Koby	WGS	3,075,888	41,113	5524	1961	1.400	merged-WGS	2,496,576	32,086	8412	3796	1.437
PTV_Clint	WGS	3,075,888	41,113	5520	1965	1.401	merged-WGS	2,496,576	32,086	8402	3806	1.438
PTV_Donald	WGS	3,075,888	41,113	5527	1958	1.400	merged-WGS	2,496,576	32,086	8413	3795	1.437
PTT/PTV_Tommy	capture	3,297,041	44,404	6563	5046	1.500	merged-capt.	2,496,576	32,086	8206	4002	1.445
PTV_7	capture	3,297,041	44,404	6694	4915	1.496	merged-capt.	2,496,576	32,086	8404	3804	1.438
PTT_Moritz	capture	3,297,041	44,404	6551	5058	1.500	merged-capt.	2,496,576	32,086	8193	4015	1.446
PTS_Bobby	capture	3,297,041	44,404	6767	4842	1.494	merged-capt.	2,496,576	32,086	8483	3725	1.434
PTE/PTV_EB176JC	capture	3,297,041	44,404	6696	4913	1.496	merged-capt.	2,496,576	32,086	8406	3802	1.437
PTV_Buttons	capture	3,297,041	44,404	6689	4920	1.496	merged-capt.	2,496,576	32,086	8400	3808	1.438
PTV_NA03450	capture	3,297,041	44,404	6699	4910	1.496	merged-capt.	2,496,576	32,086	8410	3798	1.437
PTV_8	capture	3,297,041	44,404	6693	4916	1.496	merged-capt.	2,496,576	32,086	8403	3805	1.438

**Gorillas**												
GBG_Mkubwa	WGS	2,071,756	39,991	851	353	1.947	merged-WGS	2,043,299	39,499	906	356	1.951
GBG_Kaisi	WGS	2,071,756	39,991	841	363	1.948	merged-WGS	2,043,299	39,499	896	366	1.951
GGG_Azizi	WGS	2,071,756	39,991	848	356	1.947	merged-WGS	2,043,299	39,499	902	360	1.951
GGG_Banjo	WGS	2,071,756	39,991	813	391	1.949	merged-WGS	2,043,299	39,499	867	395	1.952
GGG_Abe	WGS	2,071,756	39,991	827	377	1.948	merged-WGS	2,043,299	39,499	881	381	1.952
GGG_Tzambo	WGS	2,071,756	39,991	870	334	1.946	merged-WGS	2,043,299	39,499	924	338	1.950
GBB_Imfura	WGS	2,071,756	39,991	848	356	1.947	merged-WGS	2,043,299	39,499	903	359	1.951
GBB_Kaboko	WGS	2,071,756	39,991	850	354	1.947	merged-WGS	2,043,299	39,499	905	357	1.951
GBB_Zirikana	WGS	2,071,756	39,991	850	354	1.947	merged-WGS	2,043,299	39,499	905	357	1.951
GBG_Ntabwoba	WGS	2,071,756	39,991	842	362	1.948	merged-WGS	2,043,299	39,499	897	365	1.951
GGG_Fritz	capture	2,789,895	56,651	446	293	2.041	merged-capt.	2,043,299	39,499	876	386	1.952
GGG_Tomoka	capture	2,789,895	56,651	469	270	2.040	merged-capt.	2,043,299	39,499	896	366	1.951
GGG_Guy	capture	2,789,895	56,651	419	320	2.042	merged-capt.	2,043,299	39,499	862	400	1.953
GGG_Nikumba	capture	2,789,895	56,651	475	264	2.040	merged-capt.	2,043,299	39,499	897	365	1.951
**Orangutans**												
PAB_Buschi	WGS	2,850,916	140,215	1502	1565	4.973	merged-WGS	2,348,840	100,783	1354	1122	4.339
PPY_Napoleon	WGS	2,850,916	140,215	1433	1634	4.976	merged-WGS	2,348,840	100,783	1214	1262	4.344
PAB_Sinjo	capture	2,537,735	110,006	1327	1221	4.383	merged-capt.	2,348,840	100,783	1356	1120	4.338
PAB_Duapuluh	capture	2,537,735	110,006	1319	1229	4.383	merged-capt.	2,348,840	100,783	1347	1129	4.339
PAB_ppy10	capture	2,537,735	110,006	1331	1217	4.383	merged-capt.	2,348,840	100,783	1356	1120	4.338
PPY_Thai	capture	2,537,735	110,006	1177	1371	4.389	merged-capt.	2,348,840	100,783	1206	1270	4.345

* - WGS - whole-genome sequenced sample; capture - sample sequenced using custom-capture approach (this study); merged - WGS and capture samples analysed together to obtain overlapping sites that were used in all analysis undertaken; merged-WGS/merged-capt. - WGS/capture samples extracted from the final merged dataset

Supplemental Table S5: TMRCA estimates for nodes in the chimpanzee MSY phylogeny based on the chimpanzee reference sequence.

**MSY (PanTro4)**			
Node	N	S	TMRCA/KYA (95% HPD interval)
Chimpanzee root	19	12,127	1,225 (1,084-1,390)
PTT (1)	2	166	27.8 (22.5-33.4)
PTT (2)	17	6252	486 (428-551)
PTS/PTE/PTV (3)	16	4727	439 (387-498)
PTE/PTV (4)	13	2929	370 (326-420)
PTS (5)	3	560	82.8 (71.2-95.8)
PTE (6)	4	919	146 (128-168)
PTE (7)	3	29	4.0 (2.6-5.7)
PTV (8)	9	243	16.6 (13.7-19.6)

Numbers in parentheses in the 'Node' column refer to numbered nodes in the trees in Figure 3; for abbreviations in the same column, refer also to Figure 3; N – number of individuals; S – number of variable sites; TMRCA – time to most recent common ancestor; HPD – highest posterior density.

Supplemental Table S6: Comparison of MSY- and mtDNA-based TMRCA estimates in this study with those from other studies.

Locus	Node	N (ours)	TMRCA/KYA (95% HPD)(ours)	TMRCA/KYA (95% HPD, SD or 95% CI)* (literature estimate)	N (literature)	Data type	Ref
MSY	PPA root	4	341.1 (312.4-371.9)	500 (230-770)	7	23 SNPs/indels in ~3kb	1
				40-45 (15-135)	34	12 STRs	2
	PTR root	19	1,174.5 (1,079.7-1,275.6)	720 (370-1,070)	101	23 SNPs/indels in ~3kb	1
	PTS/PTE/PTV (3)**	16	413 (364-468)	423 (313-542)	9	7752 SNPs	3
	PTE/PTV (4)	13	360.2 (330.3-392.3)	371 (211-524)	6	7752 SNPs	3
	PTS (5)	3	77.2 (68.3-86.2)	93 (37-158)	2	7752 SNPs	3
	PTE (6)	4	151.9 (137.1-167.2)	150 (62-249)	4	7752 SNPs	3
	PTV (8)	9	13.5 (11.3-15.8)	12 (4.0-21)	2	7752 SNPs	3
	GGO root	13	104.6 (94.5-116)	142	5	630 SNPs	4
	GBB (5)	3	0.44 (0.08-0.97)	0.73	3	630 SNPs	4
	GBG/GBB (4)**	6	31.4 (26-37.1)	45	4	630 SNPs	4
	Pongo root	6	320.4 (293.3-349.7)	168 (38-375)	115	4 SNPs, 11 STRs	5
	PAB	4	9.5 (7.4-11.6)	4.2 (1.3-7.8)	40	4 SNPs, 11 STRs	5
	PPY	2	45.1 (39-51.2)	21 (9.3-35.8)	75	4 SNPs, 11 STRs	5
mtDNA	PPA root	4	307 (240.5-376.4)	410 (363-547)	34	470bp HVRI	2
				540 (430- 660)	22	complete mtDNA	6
				286 (190–406)	15	14,501bp	7
	PTR root	18	920.5 (811-1,034.3)	830 (640–1,040)	10	15,441bp	8
				1,002 (734-1,269)	24	complete mtDNA	9
				712 (458–994)	73	14,501bp	7
				1,020 (800-1,300)	4	15,599bp	10
				912 (477-1,351)	9	12 protein-coding genes (10,869bp)	3
	PTT (1)	6	323.6 (265.9-387.9)	230 (160–310)	3	15,441bp	8
				384 (235–536)	12	complete mtDNA	9
				254 (167–355)	39	14,501bp	7
	PTE/PTV (5)	11	452.2 (383.8-525.6)	410 (320–520)	7	15,441bp	8

				508 (301-715)	6	complete mtDNA	9
				376 (239–511)	21	14,501bp	7
				455 (195-719)	6	12 protein-coding genes (10,869bp)	3
	PTS (4)	3	103.7 (73.3-137.8)	80 (51–113)	13	14,501bp	7
				144 (53-242)	2	12 protein-coding genes (10,869bp)	3
	PTE (6)	4	105 (73.1-139.7)	157 (83–242)	4	complete mtDNA	9
				101 (61–149)	4	14,501bp	7
				125 (50-204)	4	12 protein-coding genes (10,869bp)	3
	PTV (7)	7	375.7 (311.8-443.5)	148 (76–223)	2	complete mtDNA	9
				289 (179–413)	17	14,501bp	7
				32 (8-61)	2	12 protein-coding genes (10,869bp)	3
	GGO root	13	1,613.9 (1,428.2-1,802.7)	1,890 (1,440-2,390)	3	15,599bp	10
				1,340	74	complete mtDNA	4
	GGG (1)	8	293.2 (231.2-358.6)	404 (284-531)	2	15,599bp	10
	Pongo root	6	2,550.8 (2,354-2,754)	3,600 (2,300-5,000)	217	323 bp from HVRI	11
				3,500 (2,310-4,750)	112	3 genes (1,355bp)	5
				3,670	76	82 SNPs from HVRI	12
				3,990 (3,010-5,090)	2	15,599 bp	10
	PAB	4	692 (592.4-798.4)	3,500 (2,310-4,750)	60	3 genes (1,355bp)	5
				3,120	64	82 SNPs from HVRI	12
	PPY	2	25.9 (8.9-47.2)	176 (72-322)	211	323 bp from HVRI	11
				178 (75-305)	52	3 genes (1,355bp)	5

Numbers in parentheses in the 'Node' columns refer to numbered nodes in the trees in Figure 3; for abbreviations in the same column, refer also to Figure 3; N – number of individuals; TMRCA – time to most recent common ancestor; HPD – highest posterior density; SD – standard deviation, CI – confidence interval. Refs.: 1 – Stone et al. 2002; 2 – Eriksson et al. 2006; 3 – Oetjens et al. 2015; 4 – Xue et al. 2015; 5 – Nater et al. 2011; 6 – Zsurka et al. 2010; 7 – Hvilsom et al. 2013; 8 – Stone et al. 2010; 9 – Bjork et al. 2011; 10 – Das et al. 2014; 11 – Arora et al. 2010; 12 – Ma et al. 2013

* - green text - TMRCA estimate in the same order as ours; blue text - literature estimate is an order higher; orange text - literature estimate is an order lower

** - comparable node chosen from the datasets

Supplemental Table S8: Filtering parameters for great ape MSY, ChrX, autosomal and mtDNA sequences.

Filter	MSY/ChrX	Autosomal	mtDNA
Indels removed	yes	yes	no
Minimum QUAL	33	33	No
Minimum depth (DP)	3	6	3
Strand bias (SP)	p<0.05	p<0.05	p<0.05
SnpGap	5	5	no
Remove heterozygous calls	Yes	No	Yes
Minimum PL difference*	No	21	No
Missing data per sample and per site	None allowed	None allowed	None allowed

* calls where the minimum difference between genotype likelihoods was less than 21 were removed.

Supplemental Table S14: TMRCA estimates for nodes in the great-ape mtDNA phylogenies based on the coding region mutation rate.

**mtDNA coding region**<br>Node	N	TMRCA/KYA (95% HPD interval)
Human root	8	206 (146-286)
Bonobo root	4	337 (237-464)
PPA (1)	3	8.3 (1.3-19.2)
Chimpanzee root	18	922 (678-1,228)
PTT (2)	2	53.4 (28.4-85.1)
PTT (1)	6	347 (248-470)
PTT/PTS (3)	5	265 (188-362)
PTE/PTV (5)	11	450 (329-606)
PTS (4)	3	125 (84-177)
PTE (6)	4	128 (86-181)
PTV (7)	7	392 (285-530)
Gorilla root	13	1,735 (1,277-2,301)
GGG (1)	8	346 (246-474)
GGG (2)	7	113 (73-164)
GBG (5)	2	4.7 (0.08-13.9)
GBB (4)	3	8.9 (1.5-19.9)
GBG/GBB (3)	5	234 (160-327)
Orangutan root	6	3,609 (2,617-4,888)
PAB	4	837 (619-1,137)
PPY	2	28.4 (11.4-50.7)

Numbers in parentheses in the 'Node' columns refer to numbered nodes in the trees in Figure 3; for abbreviations in the same column, refer also to Figure 3; N – number of individuals; TMRCA – time to most recent common ancestor; HPD – highest posterior density.

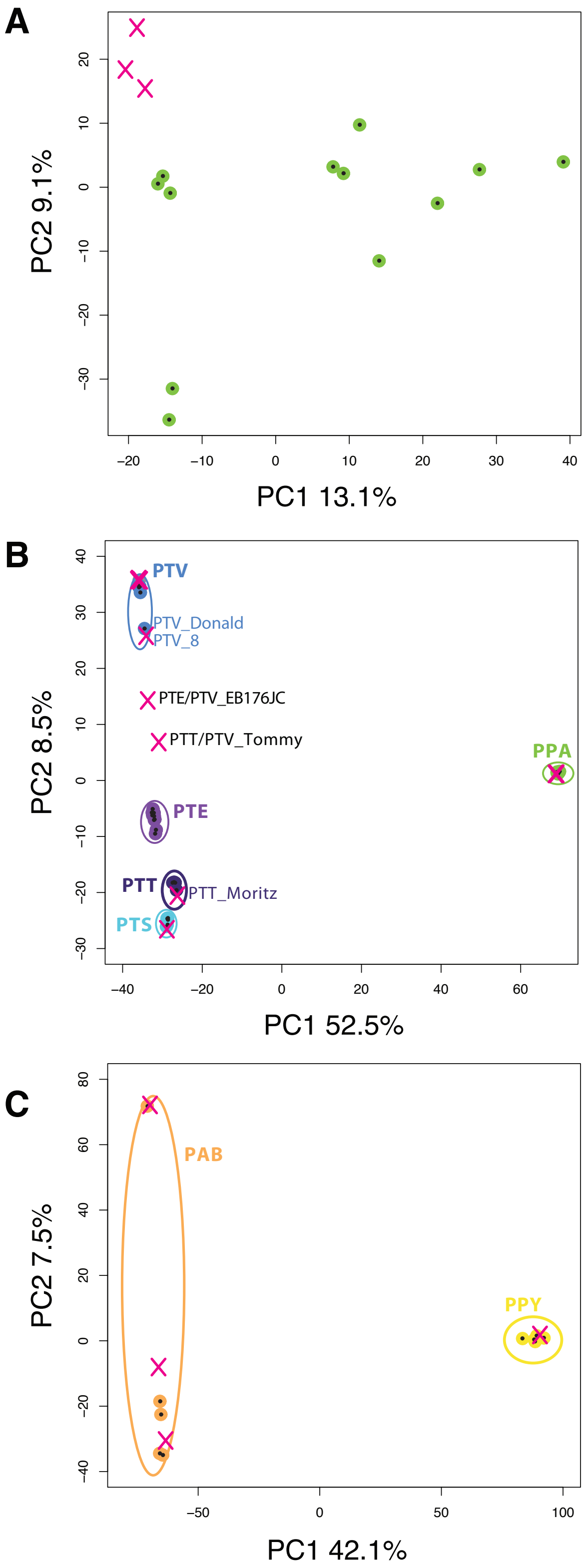

Supplemental Figure S1: PCA of autosomal SNP variation in sequenced bonobos and orangutans. PCA plots based on autosomal SNP variation in **(A)** the 3 bonobos sequenced here (crosses), plus 12 published bonobos (Prado-Martinez et al. 2013) (circles); **(B)** the same samples as (a) with the addition of 25 published chimpanzees (Prado-Martinez et al. 2013) and the 8 chimpanzees sequenced here and also represented in Figure 2A (crosses); and **(C)** the 4 orangutans sequenced here (crosses), plus 10 published individuals (Prado-Martinez et al. 2013) of known sub-species status (circles). Abbreviations for species/subspecies names are as in the legend to Figure 3.

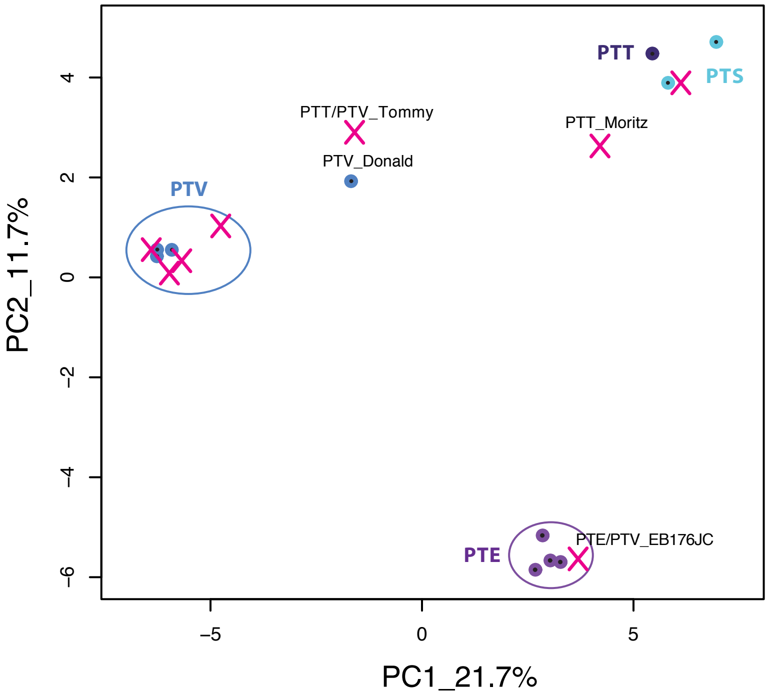

Supplemental Figure S2: PCA of X-Chromosomal SNP variation in chimpanzees. PCA plot based on X-Chromosomal SNP variation in the 8 chimpanzee males sequenced here (crosses), plus 11 published males (Prado-Martinez et al. 2013) of known sub-species status (circles). Considering the two inter-subspecies hybrids, the X Chromosome of EB176JC clusters with *P. t. ellioti* which, together with the MSY and mtDNA results, is consistent with first-generation hybrid status. The X Chromosome of the other hybrid, Tommy, lies between the PTV cluster and the group of PTT/PTS samples, and the nature of his hybrid status is less clear. Abbreviations for species/subspecies names are as in the legend to Figure 3.

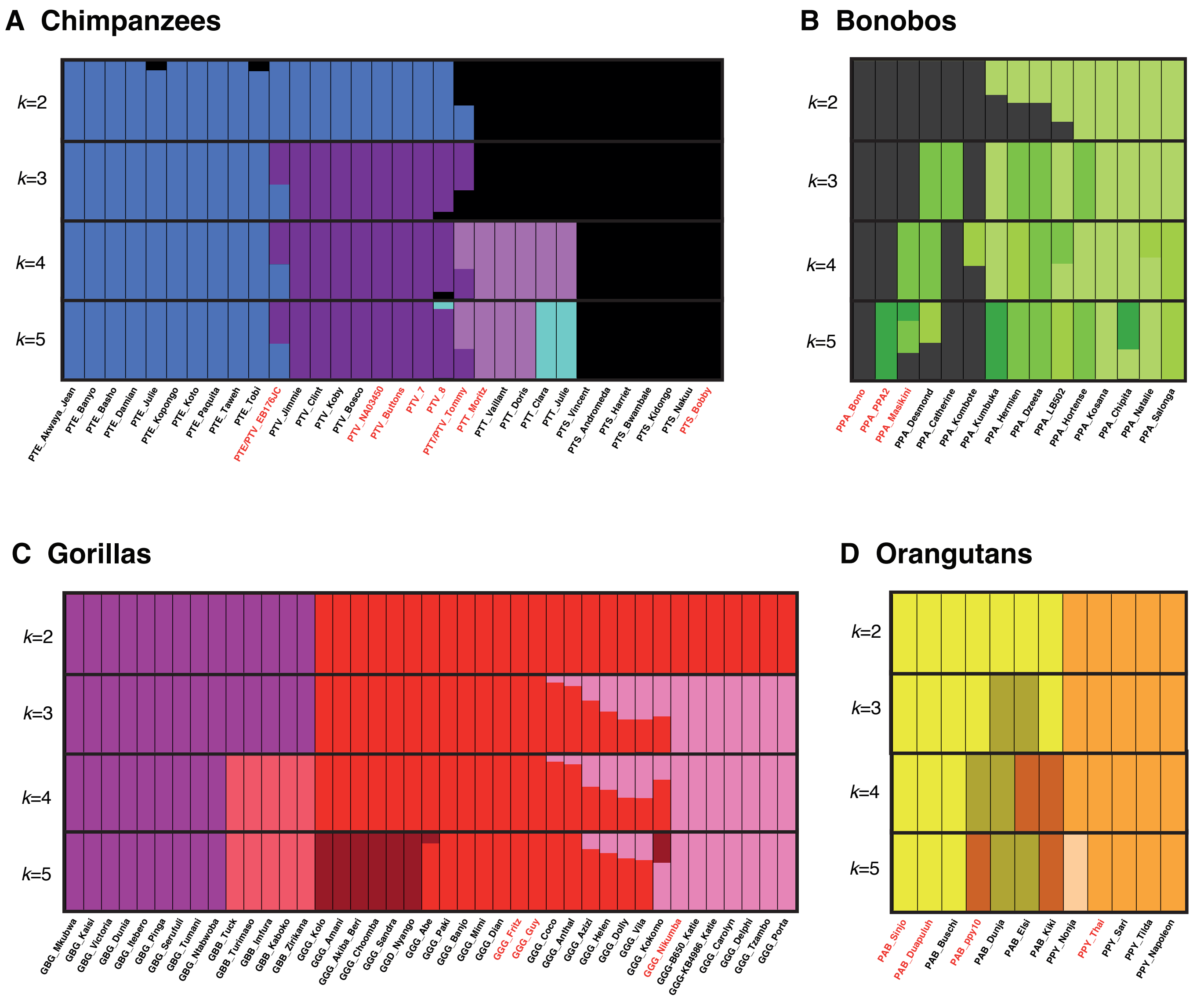

Supplemental Figure S3: Model-based estimation of ancestry in great apes using ADMIXTURE. All individuals (columns) are grouped into different clusters (*k*=2 to *k*=5, rows) coloured according to their shared genetic structure. Names of great-ape males sequenced in this study are in red.

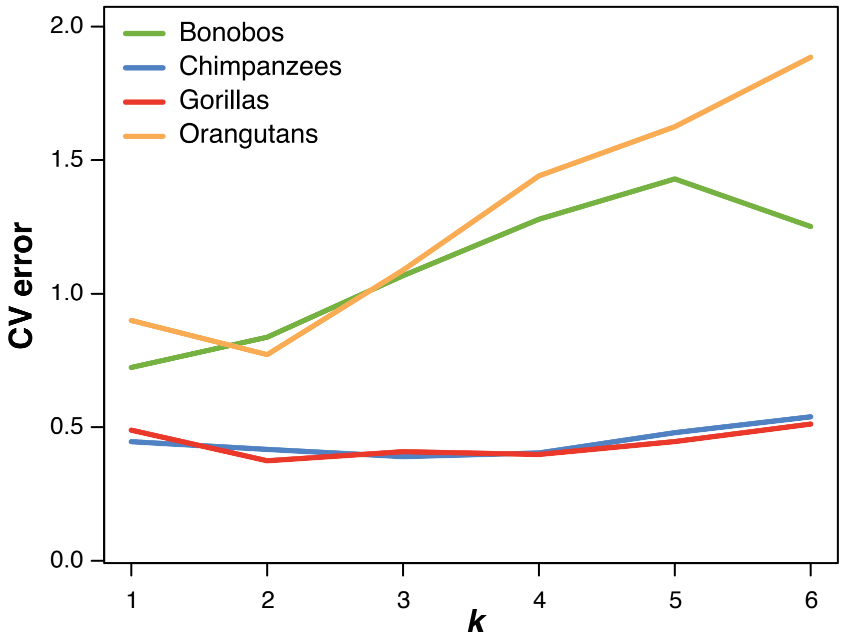

Supplemental Figure S4: Cross-validation error in ADMIXTURE analysis.

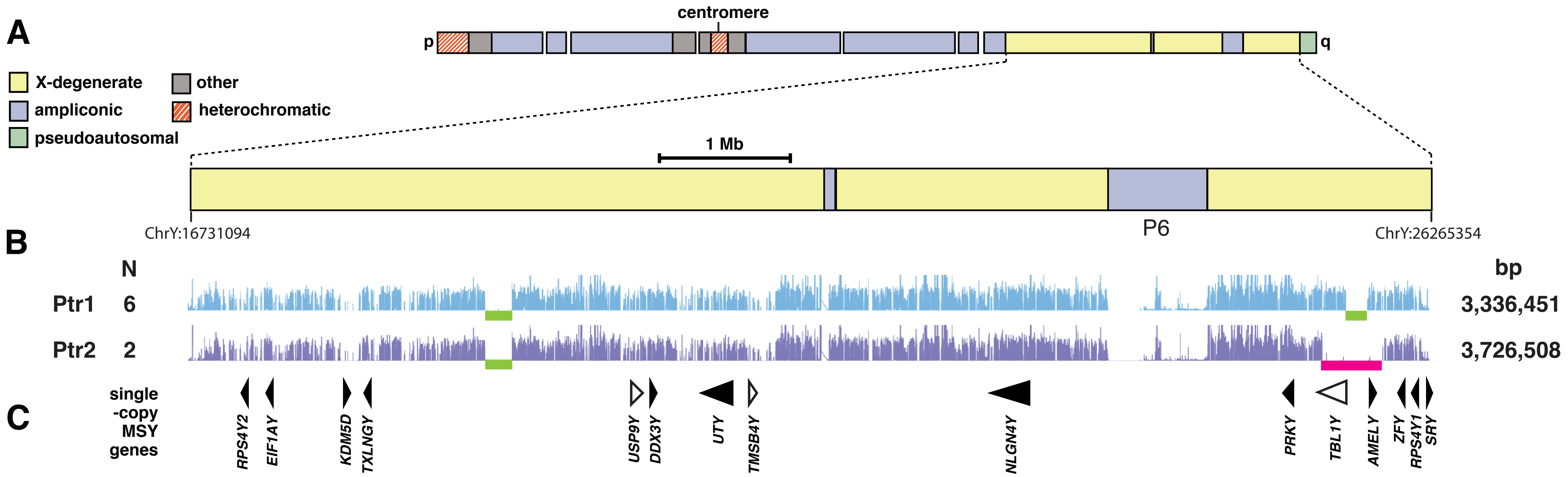

Supplemental Figure S5: Location and extent of sequenced chimpanzee MSY-orthologous regions compared to the chimpanzee reference sequence. **(A)** Schematic representation of the chimpanzee Y Chromosome (Hughes et al. 2010) showing blocks of different sequence classes. **(B)** The analysed sub-regions of chimpanzee MSY, shown as plots of read depth against chromosome position. In each plot the y-axis ranges from zero to 150 ×. Sample size (N) is given to the left, and the extent of sequence obtained (bp) to the right. Chimpanzees carry two distinct structural variant sequences (Ptr1 and 2) differing by a large deletion highlighted by a magenta bar; the gaps indicated by the green bars correspond to large blocks that are absent from the human reference sequence. The Ptr2-specific retention of a sequence block indicated in Figure 1 is not visible here since it does not exist in the chimpanzee reference sequence. **(C)** Locations of single-copy MSY genes (Hughes et al. 2010) shown as triangles (not drawn to scale) pointing in the direction of transcription. Three of these genes, functional in humans, are reported to be pseudo-genes in chimpanzees (Bellott et al. 2014), and are indicated by unfilled triangles.

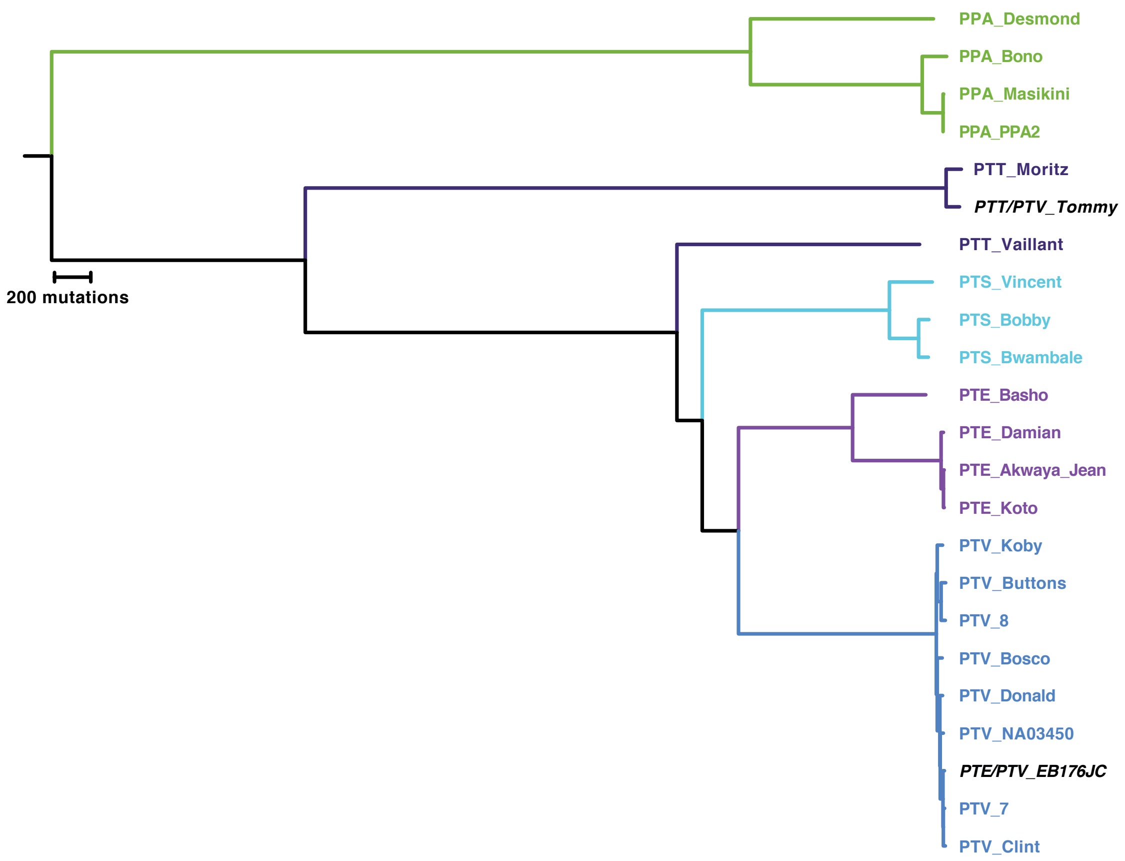

Supplemental Figure S6: MSY phylogeny for chimpanzees and bonobos combined. Species/subspecies names and names of individuals are given at the tips of branches as in Figure 3C and D.

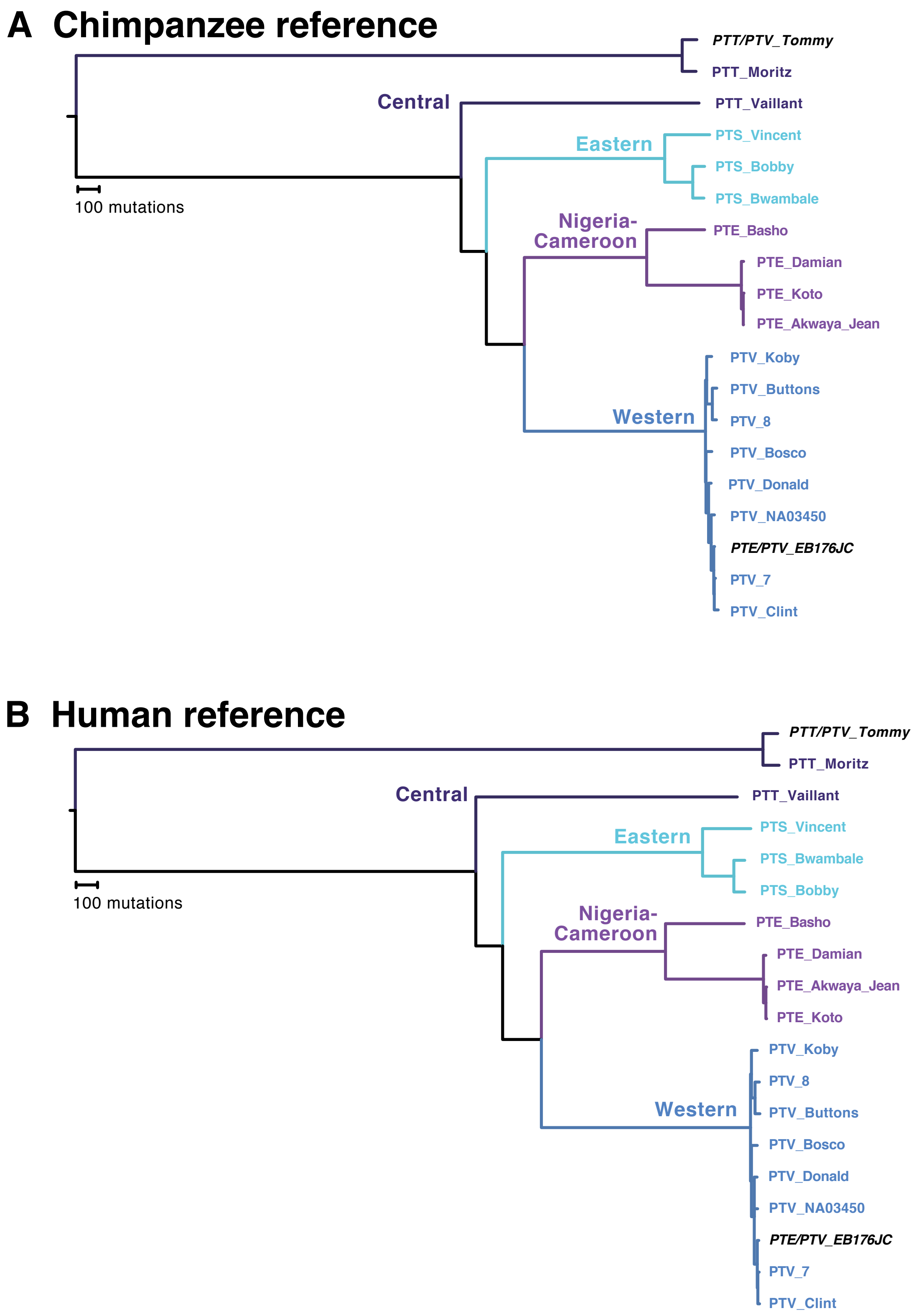

Supplemental Figure S7: Comparison of chimpanzee MSY phylogenies, based on mapping to chimpanzee and human reference sequences.
(A) Chimpanzee, and **(B)** human reference sequence mapping. Species/subspecies names and names of individuals are given at the tips of branches as in Figure 3D.

A bonobos

```
                            +PPA_Masikini
                        +---3
+-----------------------2   +PPA_PPA2
|                       |
|                       +---PPA_Bono
|
1----------------------------PPA_Desmond
|
+ancestral
```

B chimpanzees

```
                                  +PTV_7
                               +--9
                            +--8  +PTV_Clint
                            |  |
                            |  +PTE/PTV_EB176JC
                            |
                         +--7-PTV_Donald
                         |  |
                      +--6  +PTV_NA03450
                      |  |
                +-----5  +PTV_Bosco
                |     |
                |     |  +PTV_Koby
                |     +-10
                |        |  +PTV_8
                |        +-11
             +--4           +PTV_Buttons
             |  |
             |  |        +PTE_Akwaya_Jean
             |  |     +-14
             |  |  +-13  +PTE_Koto
             |  |  |  |
          +--3  +-12  +PTE_Damian
          |  |     |
          |  |     +--PTE_Basho
          |  |
          |  |    +-PTS_Vincent
+---------2  +---15
|         |       |  +PTS_Bwambale
|         |       +-16
|         |          +PTS_Bobby
|         |
|         +------PTT_Vaillant
|
1-ancestral
|
|                 +PTT/PTV_Tommy
+----------------17
                  +PTT_Moritz
```

C gorillas

```
                 +GBB_Zirika
                 |
              +--4-GBB_Imfura
              |  |
          +---3  +GBB_Kaboko
          |   |
+---------2   +GBG_Mkubwa
|         |
|         |   +GBG_Kaisi
|         +---5
|             +GBG_Ntabwoba
|
|               +GGG_Azizi
|         +-----8
|      +--7     +GGG_Nikumba
|      |  |
|      |  +----GGG_Tzambo
1------6
|      |        +GGG_Fritz
|      |  +----10
|      |  |     +GGG_Abe
|      +--9
|         |     +GGG_Banjo
|         +----11
|               +-GGG_Guy
|
+ancestral
```

D orangutans

```
                         +PAB_Duapuluh
                         |
+------------------------2     +PAB_Sinjo
|                        |  +--4
|                        +--3  +PAB_Buschi
|                           |
|                           +PAB_ppy10
|
|                         +--PPY_Thai
1-------------------------5
|                         +--PPY_Napoleon
|
+ancestral
```

E chimpanzees (PanTro4 ref.)

```
                               +PTV_NA03450
                            +--8
                            |  |  +PTE/PTV_EB176JC
                            |  +--9
                         +--7     |  +PTV_7
                         |  |     +-10
                         |  |        +PTV_Clint
                      +--6  |
                      |  |  +PTV_Donald
                      |  |
                 +----5  +PTV_Bosco
                 |    |
                 |    |  +PTV_Koby
                 |    +-11
                 |       |  +PTV_Buttons
                 |       +-12
              +--4          +PTV_8
              |  |
              |  |         +PTE_Koto
              |  |      +-15
              |  |   +-14  +PTE_Akwaya_Jean
           +--3  |   |  |
           |  |  +-13  +PTE_Damian
           |  |     |
           |  |     +-PTE_Basho
           |  |
+----------2  |    +PTS_Vincent
|          |  +---16
|          |      |  +PTS_Bobby
|          |      +-17
|          |         +PTS_Bwambale
|          |
|          +------PTT_Vaillant
|
|                +PTT/PTV_Tommy
1--------------18
|                +PTT_Moritz
|
+ancestral
```

Supplemental Figure S8: Great-ape MSY phylogenies containing named branches.
(A) Bonobos. Refer to Table S9 to find variants on specific numbered branches; **(B)** Chimpanzees mapped to human genome reference (see Table S10); **(C)** Gorillas (see Table S11); **(D)** Orangutans (see Table S12); **(E)** Chimpanzees mapped to chimpanzee genome reference (see Table S13).
